# A practical approach to replenishment optimization with extended (*R*, *s*, *Q*) policy and probabilistic models

**DOI:** 10.1038/s41598-025-32537-2

**Published:** 2025-12-19

**Authors:** Alva Presbitero, Andreas Syrén, Hagop Dippel, Lalita Awasthi, Shikhar Dev, Zhe Nie

**Affiliations:** Zalando, 10243 Berlin, Germany

**Keywords:** Replenishment optimization, Inventory management, Profit contribution, E-commerce supply chain, Probabilistic forecasting, RsQ policy, Engineering, Mathematics and computing

## Abstract

Effective inventory management is crucial for businesses operating across diverse sales channels, particularly in the dynamic e-commerce landscape. Balancing the need to minimize inventory costs while ensuring sufficient stock availability to meet fluctuating demand presents a significant challenge, further compounded by the complexities of external partnerships and distributed fulfillment networks. These factors introduce uncertainties in demand, returns, and lead times, impacting overall profitability. Recent literature has advanced optimization techniques for production and supply chain systems, often focusing on complex analytical objectives related to imperfect quality, typically solved using meta-heuristics. However, these sophisticated optimization models often rely on deterministic or simplified demand assumptions. This study presents a novel approach to replenishment optimization with a two-fold contribution. First, we aim to bridge the gap through the systematic integration of probabilistic demand forecasting with advanced inventory policy optimization techniques, bridging a critical gap between predictive modeling and practical replenishment decisions. Second, we introduce a significant extension to the classical $\left( R, s, Q\right)$ policy framework by Jansen (1996), adapting it to the specific demands of a distributed fulfillment network and highly seasonal assortments. Our Zalando E-commerce Operating System (ZEOS) Inventory Optimization Tool unifies one-shot inventory policy optimization with the predictive power of probabilistic gradient-boosting models (LightGBM). In a backtest covering the full one-year period, the system achieved an aggregate in both Gross Merchandise Value (GMV) and GMV after fulfilment costs uplift compared to the human baseline of ${\sim 22\%}$ and outperformed all classical inventory-theory baselines (Tuned $\left( s, S\right)$, periodic base-stock, and Myopic newsvendor), with average operational availability and demand fill rate maintained at ${\sim 86\%}$ and ${\sim 91\%}$, respectively. Ablation analyses show that probabilistic forecasts with a percentile-based objective yield the most robust performance. Further, horizon sensitivity analysis identifies a 12-week window as optimal for balancing responsiveness and profit stability. This research represents a pioneering effort in bridging the gap between probabilistic demand forecasting and policy optimization techniques, resulting in a practical tool that addresses the challenges of inventory management in an e-commerce setting, contributing to improved efficiency, reduced costs, and enhanced profitability.

## Introduction

Effective inventory management is a critical challenge for businesses operating across diverse sales channels, particularly in the dynamic landscape of e-commerce^1–4^. Balancing the need to minimize storage costs while ensuring sufficient stock to meet fluctuating demand requires sophisticated solutions that can accurately predict future needs and optimize replenishment strategies^5,6^. Recent academic efforts have increasingly focused on modeling complex constraints within production and supply chains, including integrating issues like imperfect production, screening errors, item deterioration, carbon emission constraints, and specific absorption rate (SAR) compliance into optimization models, often solved using optimal control theory and advanced meta-heuristics such as the Artificial Hummingbird Algorithm (AHA) and Equilibrium Optimiser Algorithm (EO)^1–4^. The complexity is further amplified in modern e-commerce by features such as highly seasonal assortments, distributed fulfillment networks, and rapid product lifecycles^7–9^. These challenges highlight the need for data-driven, risk-aware inventory control methods, especially given the ongoing interest in leveraging advanced analytics and machine learning in operations^10,11^.

While there has been considerable progress in time series forecasting and supply chain optimization fields respectively, there has historically existed a level of separation between forecasting research and supply chain optimization research^12,13^, a gap the applied practitioner inevitably must bridge in industry applications. Current practice, as Goltsos extensively discussed, often sees these domains in silos: forecasting teams deliver demand predictions (often deterministic point forecasts), which are then independently fed into optimization models that assume these forecasts as given, rather than fully integrating the probabilistic nature of demand. This disconnect might stem from different factors, including different academic backgrounds of researchers in each field, the distinct methodologies employed (e.g., statistical/machine learning for forecasting^14^ vs. operations research optimization), and the complexity of building models that can effectively bridge probabilistic uncertainties with prescriptive actions^15^. For a deeper discussion on the historical separation and the theoretical underpinnings of this phenomenon, we refer to^12^.

This paper presents the ZEOS Inventory Optimization Tool, which introduces a novel approach to replenishment optimization with two primary contributions:

1. **Bridging the probabilistic forecasting-to-optimization gap:** Our work complements the recent focus on advanced computational techniques in production control systems^1–4^. While these studies often analyze highly complex physical production phenomena (e.g., rework, SAR, and emission taxation) via analytically defined objective functions (solved using methods like Extended Pontryagin’s Maximum Principle or AHA), they typically simplify the demand modeling using deterministic or interval-based functions. We present a pioneering effort to systematically integrate probabilistic demand forecasting, generated by a LightGBM model, directly into an inventory policy optimization framework. Unlike approaches that rely solely on point forecasts, our method explicitly leverages the full distribution of future demand (via quantiles from Conformal Prediction^16,17^) as input to a simulation-assisted stochastic optimization engine^18,19^. This unique combination, not commonly found in existing literature or industry practice, offers a practical, end-to-end solution for optimizing replenishment decisions, particularly within a large-scale e-commerce environment^20,21^.
2. **Extension of the Classical **$\left( R, s, Q\right)$ **Policy for E-commerce context:** The $\left( R, s, Q\right)$ policy pioneered by Jansen^22^ is widely studied in inventory control literature (e.g.,^5,6^), allowing for a succinct representation of the decision space to a few key decision variables: review period *R* (fixed interval of time between successive inventory level checks), restock threshold *s* (predetermined inventory level at which falling below triggers a replenishment order), and restock quantity *Q* (fixed order size when a replenishment is triggered). In this work, to accommodate the specific requirements of Zalando’s distributed fulfillment network and highly seasonal assortments, we extend the classical $\left( R, s, Q\right)$ policy with additional decision variables: initial replenishment time ($t_{0}$) and quantity ($Q_{0}$), subsequent replenishment quantities (*Q*), and a termination point for the policy’s applicability ($t_{\text {limit}}$). These extensions are crucial for enabling nuanced control over replenishment timing, especially for new articles or those approaching their end-of-season lifecycle^23^, allowing the optimization to focus more on the near future where demand uncertainty is lower and decisions are more impactful.

The LightGBM gradient boosting framework was utilized due to its known computational efficiency and capacity for accurate modeling of structured data^24^, aligning with its empirically strong performance in the forecasting domain^25,26^. We demonstrate a practical union of probabilistic forecasting and supply chain optimization by showing that a gradient-free black box optimizer on a Monte Carlo simulation of the supply chain, while lacking theoretical convergence guarantees, works in practice. Finally, we validate the conclusions via backtesting on Zalando’s vast real-world operational datasets covering more than 2 million articles across a subset of around 800 merchants, along with an ablation study, sensitivity analysis and slice analysis. The ZEOS Inventory Optimization Tool offers a data-driven approach to optimize replenishment decisions, ultimately contributing to improved efficiency, reduced costs, and enhanced profitability for businesses operating in the competitive e-commerce landscape^27^.

## Methods and data

**Fig. 1:**
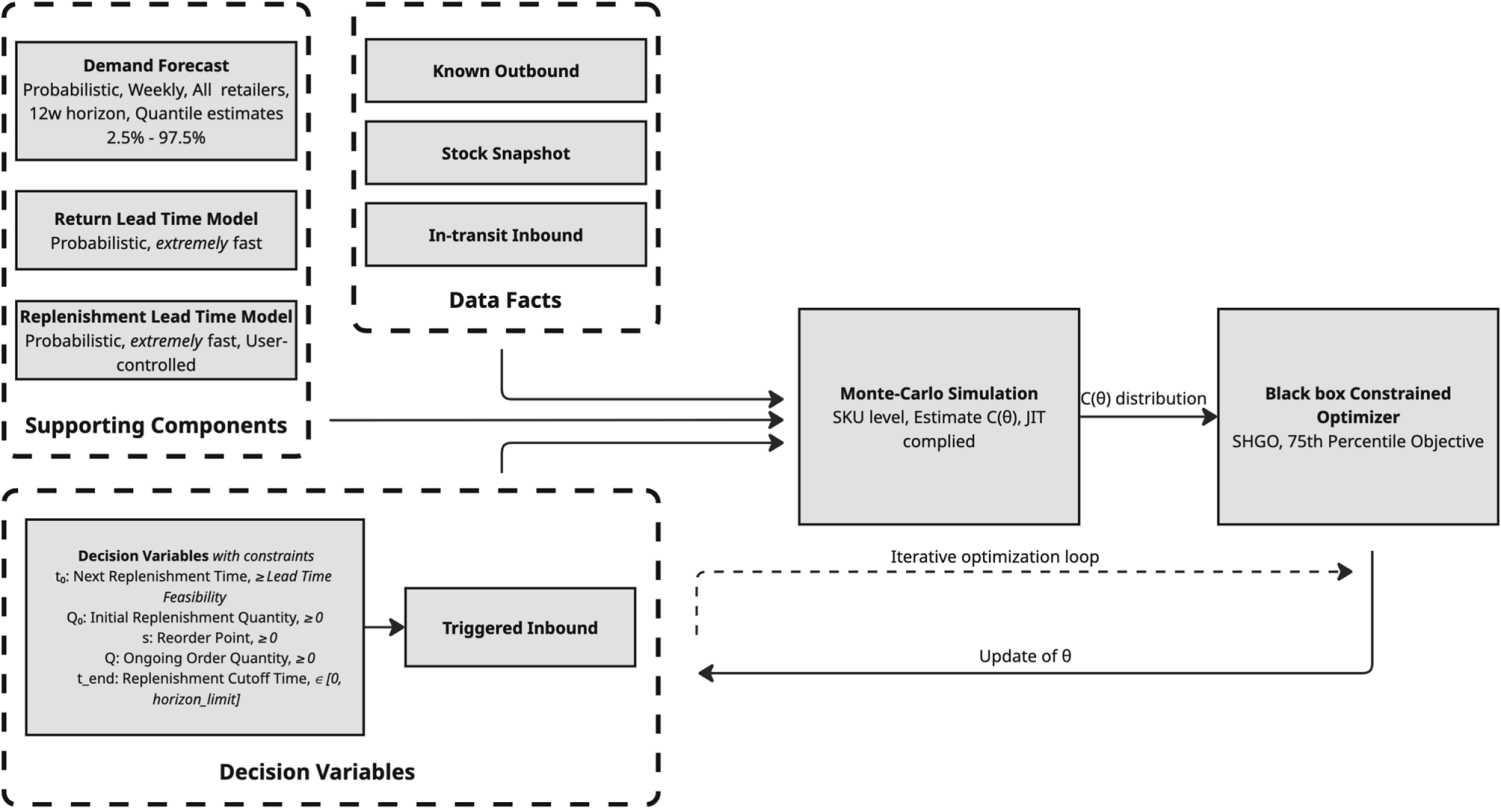
Component view of the replenishment engine. The ZEOS Inventory Optimization Tool is composed of the core replenishment engine (involving Discrete Event Simulation and stochastic optimization for policy finding) and supporting components (demand forecasting, return and replenishment lead time modeling).

The ZEOS Inventory Optimization Tool consists of the replenishment engine as core of the system and supporting components. The engine involves discrete events simulation, such as realizations of demand and return, and stochastic optimization to find the optimal replenishment policy. The supporting components involve demand forecasting, return lead time modeling and replenishment lead time modeling as summarized in Fig. 1. For a deeper dive into the supporting components, please refer to the Supplementary Material.

### Optimization method

We formulate the replenishment problem as a stochastic constrained optimization problem, with the objective of maximizing overall profit contribution. At its core, an inventory policy defines the rules or strategy governing when and how much to reorder to manage stock levels. The decision variables extend classical $\left( R, s, Q\right)$ replenishment policy parameters where we optimize five decision parameters: $t_0$, $Q_0$, *s*, *Q* and $t_{\text {limit}}$ (where *R*, the review period is fixed based on operational constraints). Crucially, our system is designed for practical adoption: we only expose the next replenishment variables $\left( t_0, Q_0\right)$ to the user through the ZEOS dashboard, managing the remaining policy parameters $\left( s, Q \text { and } t_{\text {limit}}\right)$ internally within the optimization engine. Detailed description is summarized in Table 1.

**Table 1:** Descriptions and constraints of the decision variables of a replenishment policy. Only two decision variables are actually shown to the user, the next replenishment $\left( t_0,Q_0\right)$.

Variable	Constraints	Description
$t_0$	$t_0 \ge L$ for end-to-end replenishment lead time *L*	The time at which the next replenishment will become offerable and able to satisfy demand. In order for the merchant to be able to act on this replenishment, it must be at least far enough into the future that it is feasible with respect to the merchant’s replenishment lead time.
$Q_0$	$Q_0 \ge 0$	The replenishment quantity of the next replenishment. Kickstart quantity for strategic positioning. Initial order size that provides inventory buffer before regular periodic policy activates. Enables proactive positioning against anticipated demand patterns.
*s*	$s \ge 0$	The replenish point (sometimes referred to as re-order point in literature). After $t_0$, inventory is monitored every *R* period, and new orders of size *Q* are placed when inventory position falls to or below *s*.
*Q*	$Q \ge 0$	The quantity replenished at each subsequent replenishment (triggered on review points after $t_0$ for which the stock level is below *s*).
$t_{\text {limit}}$	$t_{\text {limit}} \in [0, T]$ for some sufficiently large horizon *T*.	Order cutoff time preventing end-of-lifecycle overstock. Beyond this point, no further orders are placed regardless of inventory levels, protecting against excess inventory as demand declines toward season end.

Decision parameters (*s*, *Q*) are necessary to limit the scope of what is addressed by $(t_0,Q_0)$. If these variables are not used, the optimizer will use $(t_0,Q_0)$ to replenish inventory for the whole lifecycle of the article in one go. $t_{\text {limit}}$ is necessary for an optimizer to respond to the decline of demand at the end of an article’s lifecycle. If $t_{\text {limit}}$ is not used, *Q* tends to be underdimensioned as the optimizer attempts to reduce the impact of overstock due to a final replenishment at the end of the season.

Under a setting of complete information, an optimal replenishment policy is defined as one that minimizes the opportunity cost over the article lifecycle from the perspective of the merchant, shown as Equation (1). Equivalently, this is framed as maximizing the merchant’s profit contribution, explicitly excluding internal operational costs such as logistics, operations, and marketing.

1 $$\begin{aligned} \theta ^{**} = \text {argmin}_{\theta \in \Theta } \left[ C_{\text {holding}}(\theta ) + C_{\text {inbound}}(\theta ) + C_{\text {outbound}}(\theta ) + C_{\text {returns}}(\theta ) + C_{\text {lost\_sales}}(\theta ) \right] \end{aligned}$$

Where $\theta$ is the decision parameters $\left( t_0, Q_0, s, Q\right)$ that represent the optimal replenishment policy.

- $C_{\text {holding}}(\theta ) = f_{\text {storage}} \sum _{t=1}^{T=12} s_t$ is the cost of storage accumulated for storage fee $f_{\text {storage}}$ and stock levels $s_t$ within the 12-week simulation horizon where *t* is on a weekly cadence.
- $C_{\text {inbound}}(\theta ) = f_{\text {inbound}} \sum _{t=1}^{T=12} i_t$ is the inbound cost calculated by multiplying the cost $f_{\text {inbound}}$ to the external partner of shipping the replenished goods with the inbound $i_t$ accumulated over the simulation horizon *T*.
- $C_{\text {outbound}}(\theta ) = f_{\text {outbound}} \sum _{t=1}^{T=12} o_t$ is the cost of associated sales orders successfully placed where $f_{\text {outbound}}$ is the outbound processing cost per unit and $o_t$ is the weekly outbound that is then summed over the horizon *T*.
- $C_{\text {returns}}(\theta ) = f_{\text {return}} \sum _{t=1}^{T=12} r_t$ is the cost of returns accumulated over the horizon for returns $r_t$ where $f_{\text {return}}$ is the returns processing fee per unit.
- $C_{\text {lost\_sales}}(\theta ) = \sum _{t=1}^{T=12} (p_{\text {sales}, t} - p_{\text {purchase}})(1-\text {return rate}) d_{\text {unmet}, t}$ is the cost of lost sales derived by multiplying the difference of price $p_{\text {sales},t}$ at time step *t* by purchase price (cost of goods sold) $p_{\text {purchase}}$ with the summation of unmet demand $d_{\text {unmet}, t}$ over a 12-week horizon. This is then multiplied with the factor $(1-\text {return rate})$ to only factor in lost demand that turned into sales.

However, in real-world scenarios, complete information is unattainable for two primary reasons:

1. **Cost components are unknown in advance,** being dependent on uncertain and highly variable demand and return behavior.
2. **Cost components are counterfactual,** relying on realizations of events (e.g., demand during stock-outs or returns from unfulfilled orders) that are not directly observable.

To address these challenges, we model the replenishment problem as a simulation-assisted stochastic optimization problem, as expressed in Eq. (1). This approach is valuable because it allows for robust optimization under uncertainty, providing solutions that are expected to perform well even in the presence of noise. The stochastic formulation explicitly accounts for uncertainty by modeling key variables, such as weekly Stock Keeping Unit (SKU)-level demand (which is probabilistically forecasted using quantiles from Conformal Prediction), return lead times (sampled from fitted historical return data), and replenishment lead times (modeled using a gamma distribution to capture inherent variability), as random variables within the optimization framework. A Stock Keeping Unit (SKU) is a unique identifier for a product and its variants (e.g., size, color, or style) used to track inventory for sales and replenishment. The policy parameters are therefore determined independently for each unique product variant, explicitly ignoring dependencies on other SKUs. By representing these uncertain elements as distributions rather than fixed values, we can capture the inherent variability in the system. Moreover, the simulation-assisted approach addresses the challenge of counterfactual measurements, where outcomes like demand during stock-outs or returns on unfulfilled orders cannot be directly observed.

In this study, the optimization framework is designed as a numerical experiment where the Monte Carlo simulation serves to rigorously evaluate replenishment policies across diverse, probabilistic demand scenarios. The fundamental structure of the optimization loss function (Eq. 1) is explicitly aligned with critical merchant financial and operational objectives. By minimizing the total cost, which comprises holding, inbound, outbound, returns processing, and lost sales costs, the objective function directly proxies the financial success of the merchant by maximizing their profit contribution, hence minimizing costs. The outcomes of this numerical evaluation are assessed not just by the cost objective, but by practical, reported business Key Performance Indicators (KPIs) such as weekly availability rate, demand fill rate, and Gross Merchandise Value (GMV) and GMV after fulfillment costs (margin). By simulating stock trajectories and computing associated costs under uncertainty, the experiments rigorously quantify the trade-offs between the costs (e.g., increased holding cost vs. reduced lost sales cost). This direct alignment ensures that the optimized policy parameters–which show how to improve service levels while maintaining or enhancing profitability–provide immediately actionable insights for e-commerce inventory management.

### Simulation method

We use available observations to construct a Monte Carlo simulation. To evaluate the cost distribution $C(\theta )$ corresponding to a candidate replenishment policy $\theta$, each simulation run samples from estimated distributions of:

1. Weekly SKU-level demand, based on a probabilistic forecast.
2. Return lead times, drawn from fitted historical return data.
3. Replenishment lead times, parameterized by user-specified base values.

The simulation begins with an initial state defined by the current stock level and unreturned orders within the return window. A Discrete Event Simulation (DES) then models the week-by-week evolution of inventory over a 12-week horizon, driven by the following sequence of events and state updates:

- **Initial state (start of week):** The simulation tracks the current on-hand inventory (physical stock available) and in-transit inventory (orders placed but not yet received). Potential returns are implicitly modeled based on historical outbound sales and a return lead time distribution.
- **Inbound & return processing (intra-week):** To distribute events across the week and simplify the assumption that inbounds can arrive at any point within a week, a portion (half) of the current week’s scheduled inbound arrivals and expected returns are added to the on-hand inventory before demand fulfillment. The remaining portion (half) of the current week’s inbounds and returns are added after demand fulfillment.
- **Demand realization:** A weekly SKU-level demand value is sampled from the probabilistic forecast distribution, representing the potential customer demand for that week.
- **Sales fulfillment:** The simulated demand is then fulfilled from the available on-hand inventory. If demand exceeds available stock, a stock-out occurs, and the unmet demand contributes to lost sales costs.
- **End-of-week stock calculation:** All movements (initial stock, intra-week inbounds/returns, sales) are combined to calculate the $\text {stock}_t$ (stock at time *t*) representing the end-of-week on-hand inventory.
- **Replenishment decision & order placement:** At predefined review points (every *R* weeks, or at the optimized $t_0$ for the initial order), the replenishment policy is evaluated based on the calculated stock at time *t*. The policy parameters include $t_0$ (next replenishment time index, an optimized initial order time), $Q_0$ (Initial Replenishment Quantity, the quantity ordered at $t_0$), *s* (Reorder Point, where if $\text {stock}_t$ falls to/below *s* at a review point and no inbound is in transit, an ongoing order is triggered), *Q* (Ongoing Order Quantity, the fixed quantity ordered when ongoing replenishment is triggered), *R* (Review Period, a fixed frequency for ongoing reviews), and $t_{\text {limit}}$ (Time Limit for Initial Order, the last index for $Q_0$ placement). If triggered, an order for *Q* (or $Q_0$) is placed. This order immediately enters the in-transit inventory and is scheduled to arrive in a future week after its sampled lead time.
- **Shipment arrival (for future weeks):** Orders already in transit from previous weeks are modeled to arrive in future weeks once their sampled replenishment lead time elapses. (Note: The effect of these arrivals for the current week is accounted for earlier in the intra-week processing).
- **State update & cost accumulation:** At the end of each simulated week, all inventory levels (on-hand, in-transit) are updated. Weekly cost components (storage, inbound, outbound, returns, lost sales) are recorded. Total costs for the entire 12-week horizon are calculated at the simulation’s end, based on these accumulated weekly components, often applying an exponential decay factor.

**Fig. 2:**
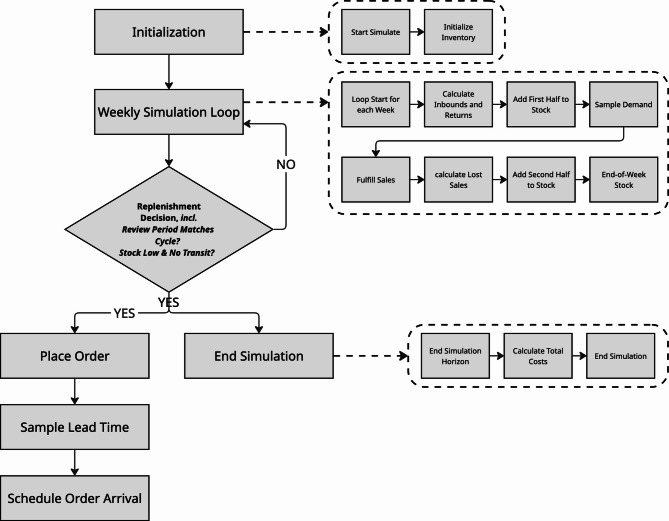
Simplified illustration of the Discrete Event Simulation state evolution. Each sample in the Monte Carlo simulation represents an execution of the DES. At the end of the simulation, the costs as described in Eq. (1) are computed and added up, yielding a sample from $C(\theta )$.

A simplified illustration of the DES state evolution is provided in Fig. 2.

This simulation generates a distribution of costs $C(\theta )$, incorporating critical trade-offs across components such as storage, fulfillment, returns, and lost sales. Rather than using the expected value $\mathbb {E}[C(\theta )]$ as the optimization objective, we select a robust percentile-based criterion. Specifically, the 75th percentile of the cost distribution. This conservative approach mitigates risk by reducing sensitivity to extreme but rare events (e.g., unexpected demand spikes), thus avoiding overly aggressive replenishment decisions. The choice of the 75th percentile reflects a risk-aware design, aiming for solutions that perform reliably in the presence of noise, and is grounded in established supply chain risk management principles. Following^28^, optimizing higher percentiles provides a tractable approximation to Conditional Value at Risk (CVaR), particularly appropriate for inventory systems where tail risks have asymmetric business impact. This choice aligns with industry practice, where inventory systems typically target 75-95% service levels^29^, and reflects realistic managerial risk preferences demonstrated in supply chain decision-making experiments^30^. The 75th percentile strikes an optimal balance between risk protection and computational stability for our 500-simulation Monte Carlo framework, as percentiles above 80–85% can lead to optimization instability in finite-sample stochastic programming contexts^31^. Although we have done some initial assessments for a cost percentile of 50% and 90% respectively (see more details in the Supplementary Material) where we found that performance gains plateau beyond the 75th percentile, this robustness parameter is tunable and sensitivity analysis across alternative percentiles will be explored in future work.

### Replenishment engine

The Replenishment Engine maps a policy $\theta$ to a distribution over costs, $C: \theta \rightarrow \mathcal {P}(\mathbb {R})$ as a function of replenishment policy $\theta$ to some distribution $C(\theta ) \in \mathcal {P}(\mathbb {R})$ where $\mathcal {P}(\mathbb {R})$ denotes the set of probability distributions on $\mathbb {R}$. This mapping allows the optimizer to capture the full range of potential outcomes under uncertainty. Specifically, we use the practical optimization problem represented in Eq. (2), where the goal is to identify an optimal replenishment policy that minimizes the cost objective while accounting for the stochastic nature of demand, returns, and shipment lead times.

2 $$\begin{aligned} \theta ^{*} = \text {argmin}_{\theta \in \Theta } \hat{Q}_{75}[C(\theta )] \end{aligned}$$

Where $\theta \in \Theta$ is a feasible replenishment policy, $\hat{Q}_{k}$ is the *k*th percentile function, and $C(\theta )$ is the estimated cost for a given policy.

**Fig. 3:**
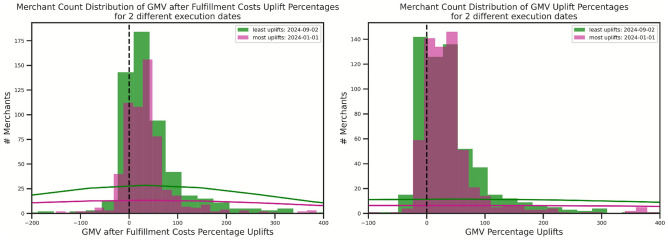
Distribution of GMV and GMV after Fulfillment Costs Uplifts (September 2024 and January 2024). Histograms illustrate the distribution of uplifts for execution dates corresponding to the least (September 2024) and most (January 2024) observed uplifts, showing over $70\%$ of merchants with positive uplifts under $100\%$ model adoption.

In practice, to solve for the optimal policy $\theta ^*$, we minimize the 75th percentile of the simulated cost distribution using a gradient-free black-box constrained optimizer, specifically SHGO (Simplicial Homology Global Optimization)^32^. The optimal policy is solved independently for each simple SKU, under the assumption of ‘no cannibalization / affinity’, meaning the policies for different SKUs do not influence each other. Figure 3 presents a component view of the complete system used to solve for $\theta$.

### Baseline comparators and evaluation framework

To evaluate the performance of the proposed machine-learning–enhanced replenishment methodology, we designed a comprehensive evaluation framework that benchmarks our model against both canonical inventory-control policies from operations-research theory and human decision-making baselines. This multi-baseline design isolates the contribution of the machine-learning component by ensuring methodological parity in optimization procedures, cost structures, and information availability across all approaches.

**Classical inventory-theory baselines.** Three canonical policies were implemented to span the theoretical spectrum of inventory-control paradigms:

- **Continuous-review **$\left( s,S\right)$ **policy** – provides real-time inventory monitoring with reorder point *s* and order-up-to level *S*, representing the theoretical upper bound for systems with continuous visibility and unlimited review frequency^5^.
- **Periodic base-stock policy** – orders up to a fixed target level *S* at every review period *R*, regardless of current stock. It aligns with business environments that operate on fixed review cycles (e.g., supplier or budget-constrained B2B settings) rather than continuous triggers^6^.
- **Myopic newsvendor policy** – solves a single-period optimization problem at each review epoch, balancing overage-and-underage costs for the immediate period while ignoring multi-period effects. Although theoretically suboptimal over multiple periods, it remains empirically competitive due to its simplicity and short-term adaptability^33,34^.

**Extended **$\left( R, s, Q\right)$ **Policy.** Our primary contribution extends classical $\left( R, s, Q\right)$ inventory theory through data-driven parameter optimization. Instead of assuming fixed demand distributions or simplified cost formulations, the model uses probabilistic demand forecasts and stochastic simulation to optimize the decision variables $\left( t_0, Q_0, s, Q, t_{\text {limit}}\right)$. This formulation explicitly incorporates forecast uncertainty, lead-time variability, and detailed cost components (holding, stockout, and transaction costs) while satisfying real-world business constraints such as review frequency and minimum order quantities.

**Human-decision benchmark.** The human baseline represents merchant-generated replenishment decisions derived from operational data. Merchants use complete historical and real-time information–inventory positions, pipeline orders, supplier reliability, seasonal context, promotions, and qualitative market intelligence–to make judgment-based, adaptive decisions. Review frequency is variable and determined by workload, supplier constraints, and strategic priorities rather than by fixed cycles. This baseline captures expert intuition and contextual reasoning unavailable to purely algorithmic policies.

For a summary of the differences in the evaluation framework used, refer to Table 2 in the Supplementary Material.

The performance of all computational policies (including the classical inventory-theory baselines) was evaluated using the following key financial and operational metrics, which were then used to quantify the uplift of each policy relative to the human benchmark decision:

- **Gross merchandise value (GMV):** The total value of merchandise sold, serving as a primary indicator of revenue performance.
- **GMV after fulfillment costs:** GMV adjusted for operational expenditures (e.g. holding costs, outbound shipping, returns processing, and inbound costs), providing a measure of margin preservation and cost-effectiveness.
- **Weighted weekly availability rate:** The percentage of time a product is in stock and available for sale, weighted by the potential revenue of demanded units (calculated as demand $\times$ price after discount), reflecting the system’s ability to prioritize high-value products and minimize stock-outs.
- **Weighted demand fill rate:** The proportion of customer demand fulfilled from available stock, weighted by the potential revenue of demanded units (calculated as demand $\times$ price after discount), measuring the success of the replenishment strategy in meeting actual demand for high-value products and minimizing lost sales.

### Data

The ZEOS Inventory Optimization Tool leverages a comprehensive multi-source data ecosystem from enterprise-scale e-commerce infrastructure, including transactional sales records capturing historical demand patterns and commission structures, enriched product metadata providing SKU hierarchies and categorical attributes, daily availability and pricing data incorporating promotional campaigns and discount structures, and commercial calendar information for seasonality modeling.

The Machine Learning Engineering architecture, which facilitates the real-time data ingestion, feature store management, and deployment of the probabilistic forecasting model, is further detailed in a dedicated engineering article^27^.

The integrated pipeline constructs both static and dynamic feature sets spanning time-invariant product characteristics (merchant identifiers, product hierarchies, brand classifications) and temporal variations through pricing histories, availability indicators, and campaign flags. Sophisticated lag features spanning 1-8 weeks complement rolling statistical measures over 4-8 week windows, alongside calendar-based encodings for seasonality capture. For the demand forecast model, the training/validation employs robust temporal splitting with 62-week training windows and 132-week feature engineering pipelines, implementing 4-fold cross-validation with walk forward fashion to prevent temporal contamination. Conformal calibration generates 39 quantile estimates (2.5–97.5%) at 2.5% increments for uncertainty quantification. The optimization engine employs a two-stage Monte Carlo approach: 500 trajectory samples for SHGO-based parameter optimization and 5000 trajectory samples for detailed quantile-based performance metrics (fixed random seeds for reproducibility), incorporating cost components spanning holding, transaction, processing, and penalty costs. The 75th percentile cost objective guides optimization toward robust solutions that perform well under adverse demand conditions. Anti-leakage guardrails enforce temporal boundaries ensuring no training data exceeds freeze dates, with future information validated against cutoff dates and deterministic processing through environment-controlled parameters and date-based partitioning for reproducible optimization decisions.

## Results and discussion

The replenishment engine serves as the central component of an inventory management framework within Zalando Fulfillment Solutions (ZFS)^27^, providing precise, policy-driven replenishment recommendations at the SKU level. These recommendations, generated daily, assist ZFS partners in optimizing their inventory across both wholesale and e-commerce platforms.

To evaluate the effectiveness of the replenishment engine, a backtest analysis was conducted using historical data. Considering the inherent volatility and seasonality of e-commerce, the backtest spanned a continuous 12-month period (October 2023 to September 2024) to thoroughly assess the system’s month-to-month consistency. Unless otherwise specified, single-date experiments (ablations, horizon and percentile sensitivity) were computed at execution date = 2024-09-02, the least-uplift date within the year, to provide a conservative assessment of robustness. The backtest involved generating model-driven replenishment recommendations and comparing them against historical human decisions. Also, the backtest assumed demand estimation through extrapolation from size distribution, serving as a ground truth to evaluate the performance. To avoid sample selection bias, we incorporated all merchants (approximately 800 merchants) with available articles to evaluate the backtest. For clarity throughout this article, all references to Weekly Availability Rate and Demand Fill Rate refer to their weighted counterparts, as defined in the section “Baseline comparators and evaluation framework”.

### Model performance compared to baseline comparators

Table 2 presents a backtest comparison between our Extended $\left( R, s, Q\right)$ policy and three analytical baselines–tuned $\left( s, S\right)$, periodic base-stock, and myopic newsvendor–compared to historical human decisions. Results were computed utilizing data spanning a full year from October 2023 to September 2024 (corresponding to 12 monthly execution dates). All models were compared with the human baseline and assessed with a 6 weeks backtest horizon.

**Table 2:** Comparative Performance of Baseline Replenishment Policies. Evaluation of the Extended $\left( R, s, Q\right)$ policy against tuned $\left( s, S\right)$, periodic base-stock, myopic newsvendor, and historical human decisions under identical data, cost, and stochastic simulation settings. All results are reported covering 1 full year, from October 2023 to September 2024, with a 6-week backtest horizon. Metrics include GMV uplift, GMV after fulfillment costs uplift, proportion of merchants with positive GMV uplifts, weighted weekly availability, weighted demand fill rate, and uplifts with respect to the human benchmarks (pp = percentage points). Bracketed values denote $95\%$ confidence intervals.

Policy	GMV Uplift wrt Human	Uplift of GMV After Fulfillment costs wrt Human	% Merchants with Positive GMV Uplifts	Weighted Weekly Availability Rate	Weighted Demand Fill Rate	Weighted Weekly Availability rate uplift wrt Human	Weighted Demand fill rate uplift wrt Human
Our Extended (*R*, *s*, *Q*)	22.11% [20.44, 23.92]	21.95% [20.03%, 24.03%]	$75.36\%$ [74.34, 76.34]	86.40 [86.18, 86.63]	91.14 [91.00, 91.28]	$+33.63\%$ $[+32.70, +34.59]$ $+21.75\text {pp}$ $[+21.29, +22.19]$	$+23.63\%$ $[+22.99, +24.26]$ $+17.42\text {pp}$ $[+17.05, +17.78]$
Tuned (*s*, *S*)	13.39% [11.91%, 14.87%]	14.80% [13.13%, 16.54%]	$63.35\%$ $[62.21\%, 64.48\%]$	77.80 [77.45, 78.16]	85.31 [85.04, 85.55]	$+18.65\%$ $[+17.83\%, +19.48\%]$ $+12.23\text {pp}$ $[+11.76, +12.69]$	$+14.35\%$ $[+13.81\%, +14.92\%]$ $+10.71\text {pp}$ $[+10.34, +11.08]$
Base-Stock	12.50% [11.11%, 14.02%]	13.89% [12.29%, 15.65%]	$63.34\%$ $[62.20\%, 64.47\%]$	77.32 [76.95, 77.70]	85.11 [84.85, 85.37]	$+17.99\%$ $[+17.18\%, +18.85\%]$ $+11.79\text {pp}$ $[+11.35, +12.2]$	$+14.19\%$ $[+13.67\%, +14.74\%]$ $+10.57\text {pp}$ $[+10.21, +10.92]$
Myopic Newsvendor	5.07% [3.33%, 6.78%]	5.60% [3.84%, 7.36%]	$54.98\%$ $[53.81\%, 56.15\%]$	73.07 [72.66, 73.49]	80.48 [80.16, 80.78]	$+11.61\%$ $[+10.84\%, +12.37\%]$ $+7.60\text {pp}$ $[+7.16, +8.05]$	$+8.10\%$ $[+7.56\%, +8.61\%]$ $+6.03\text {pp}$ $[+5.66, +6.38]$

Our Extended $\left( R, s, Q\right)$ policy achieved a GMV uplift of $\mathbf {22.11\%}$, outperforming the next-best algorithmic baseline, Tuned $\left( s, S\right)$ $\left( 13.39\%\right)$, by 8.72 percentage points (pp). The uplift in GMV after fulfillment costs $\left( 21.95\%\right)$ mirrored this trend, confirming that financial benefits persist after operational deductions. The policy maintained the highest weighted weekly availability $\left( 86.40\%\right)$ and weighted demand fill rate $\left( 91.14\%\right)$ among all algorithms, translating to an uplift of $\mathbf {+21.75\text { pp}}$ in availability and $\mathbf {+17.42\text { pp}}$ in fill-rate relative to human operations. This hierarchy of results reinforces the conceptual premise that while classical models are constrained by their reactive nature and limited optimization dimensions, the strategic positioning capability of Extended $\left( R, s, Q\right)$ allows it to establish superior initial inventory states and utilize more optimization parameters effectively. All policies operate under identical stochastic conditions (variable lead times, demand uncertainty, asymmetric costs), but Extended $\left( R, s, Q\right)$’s advantage stems from its ability to proactively optimize strategic timing ($t_0$) and initial quantities ($Q_0$) rather than purely reacting to inventory depletion like classical approaches.

### Overall backtest performance with respect to the human baseline

Table 3 presents a detailed summary of the Replenishment Engine’s performance against benchmarks (“human decisions”) across varying backtest horizons (4, 6, and 8 weeks), utilizing data spanning a full year from October 2023 to September 2024 (corresponding to 12 monthly execution dates).

**Table 3:** Summary of the Extended $\left( R, s, Q\right)$ Policy’s Performance by Backtest Horizon. The metrics are derived from backtest runs covering a full year (October 2023 to September 2024) and are presented as uplift relative to the human benchmark decision. Values in brackets denote $95\%$ confidence intervals. pp is percentage points.

Backtest horizon	GMV Uplift wrt Human	Uplift of GMV After Fulfillment costs wrt Human	% Merchants with Positive GMV Uplifts	Weighted Weekly Availability Rate	Weighted Demand Fill Rate	Weighted Weekly Availability rate uplift wrt Human	Weighted Demand fill rate uplift wrt Human
4 weeks	$19.45\%$ $[17.97\%, 21.09\%]$	$19.66\%$ $[17.90\%, 21.47\%]$	$73.43\%$ $[72.40\%, 74.44\%]$	82.85 [82.60, 83.11]	88.14 [87.97, 88.30]	$+31.51\%$ $[+30.55, +32.51]$ $+19.85\text {pp}$ $[+19.38, +20.29]$	$+21.31\%$ $[+20.68, +21.93]$ $+15.48\text {pp}$ $[+15.12, +15.85]$
6 weeks	$22.11\%$ [20.44, 23.92]	$21.95\%$ $[20.03\%, 24.03\%]$	$75.36\%$ [74.34, 76.34]	86.40 [86.18, 86.63]	91.14 [91.00, 91.28]	$+33.63\%$ $[+32.70, +34.59]$ $+21.75\text {pp}$ $[+21.29, +22.19]$	$+23.63\%$ $[+22.99, +24.26]$ $+17.42\text {pp}$ $[+17.05, +17.78]$
8 weeks	$21.90\%$ $[20.19\%, 23.83\%]$	$21.46\%$ $[19.47\%, 23.63\%]$	$75.38\%$ $[74.37\%, 76.37\%]$	87.62 [87.39, 87.85]	92.27 [92.13, 92.40]	$+32.58\%$ $[+31.70, +33.52]$ $+21.53\text {pp}$ $[+21.07, +21.98]$	$+23.54\%$ $[+22.93, +24.16]$ $+17.58\text {pp}$ $[+17.22, +17.94]$

To quantify uncertainty, $95\%$ confidence intervals were computed for all aggregated metrics in Table 3. For ratio- and mean-based indicators (e.g., GMV uplift, GMV after fulfilment costs uplift, weekly availability rate, and demand fill rate), confidence intervals were estimated using nonparametric merchant-level bootstrapping with 2,000 resamples. In this procedure, merchants were treated as independent observational units, resampled with replacement, and the relevant metric was recalculated for each bootstrap sample; the 2.5th and 97.5th percentiles of the resulting distribution defined the interval bounds. For proportion-based measures (e.g., percentage of merchants with positive GMV uplift), Wilson score intervals were used to obtain statistically robust bounds even for finite merchant counts. This approach ensures that the reported uncertainty reflects cross-merchant variability and provides a conservative estimate of the model’s performance stability across the tested backtest horizons.

As Table 3 is shown, the model consistently generated positive GMV uplifts, with a median ranging from $19.45\%$ to $22.11\%$ across the assessed backtest horizons. Importantly, GMV after fulfillment costs also exhibited substantial positive uplifts ($19.66\%$ to $21.95\%$). This indicates that revenue gains were not negated by disproportionate operational expenditures, demonstrating the algorithm’s effectiveness in mitigating overstock scenarios through the strategic integration of fulfillment costs into its optimization objective function. A significant majority of all backtested merchants ($73.43\%$ to $75.38\%$) experienced positive GMV uplifts in the span of 1 year.

The replenishment engine shows an improvement from a computational backtest strategy over the human benchmark from October 2023 to September 2024. While the replenishment engine’s prediction horizon spans 12 weeks, its performance metrics were evaluated over a 4, 6 and 8-week horizons. This design inherently places the backtest at a disadvantage compared to human decision-making, as human operators can dynamically adjust replenishment strategies on a weekly basis, a flexibility not available to the recommendation engine’s one-shot optimization. The evaluation indicates a projected positive uplift in Gross Merchandise Value (GMV) for approximately $\mathbf {75\%}$ of our ZFS (Zalando Fulfillment Solutions) merchants, encompassing over 800 individual entities. Operationally, the recommender achieved average weekly availability rates of approximately $\mathbf {80\%}$ and demand fill rates exceeding $\mathbf {85\%}$ during backtesting, reflecting a performance improvement of approximately $\mathbf {30\%}$ relative to “human decisions”. These findings strongly support the hypothesis that the proposed algorithm can substantially improve business performance for ZFS merchants, underscoring the potential for considerable financial gains and enhanced operational efficiency.

The operational metrics, specifically weekly availability rates (82.85–87.62%) and demand fill rates (88.14–92.27%), underscore robust performance. Weekly availability rate directly reflects how consistently products are in stock and ready to be sold. A higher availability rate means fewer instances of a product being out of stock. Demand fill rate on the other hand is a direct measure of how well the replenishment strategy is meeting actual customer demand. A high demand fill rate indicates that the replenishment system is successfully ensuring that products are available when customers want them, minimizing lost sales due to stockouts. These figures represent significant improvements over human benchmarks, with availability rate uplifts of 19.85–21.75 percentage points and demand fill rate uplifts of 15.48–17.58 percentage points (or $21.31\%$–$23.63\%$ in percentage terms). The backtesting result consistently showed GMV and GMV after fulfillment costs uplifts exceeding $5\%$ on average, with over $70\%$ of merchants experiencing positive uplifts. The demand fill rate and weekly availability rate targets of $>80\%$ and $>75\%$, and the consistent positive GMV uplifts observed across the 12-month backtest horizon directly confirm its temporal stability. Representative histograms, as shown in Fig. 3, illustrate GMV and GMV after Fulfillment Costs uplifts for the least (September 2024) and most (January 2024) observed uplifts dates. Both histograms show distributions primarily to the right of zero, indicating that over $75\%$ of merchants experience positive uplifts in both periods: month with the least vs that of the most uplifts. Comparison between the least uplifts date and most uplifts date (PC uplifts of $75.3\%$ versus $75.8\%$) clearly shows that the replenishment engine is robust to drive positive uplift for merchants across the whole year.

**Fig. 4:**
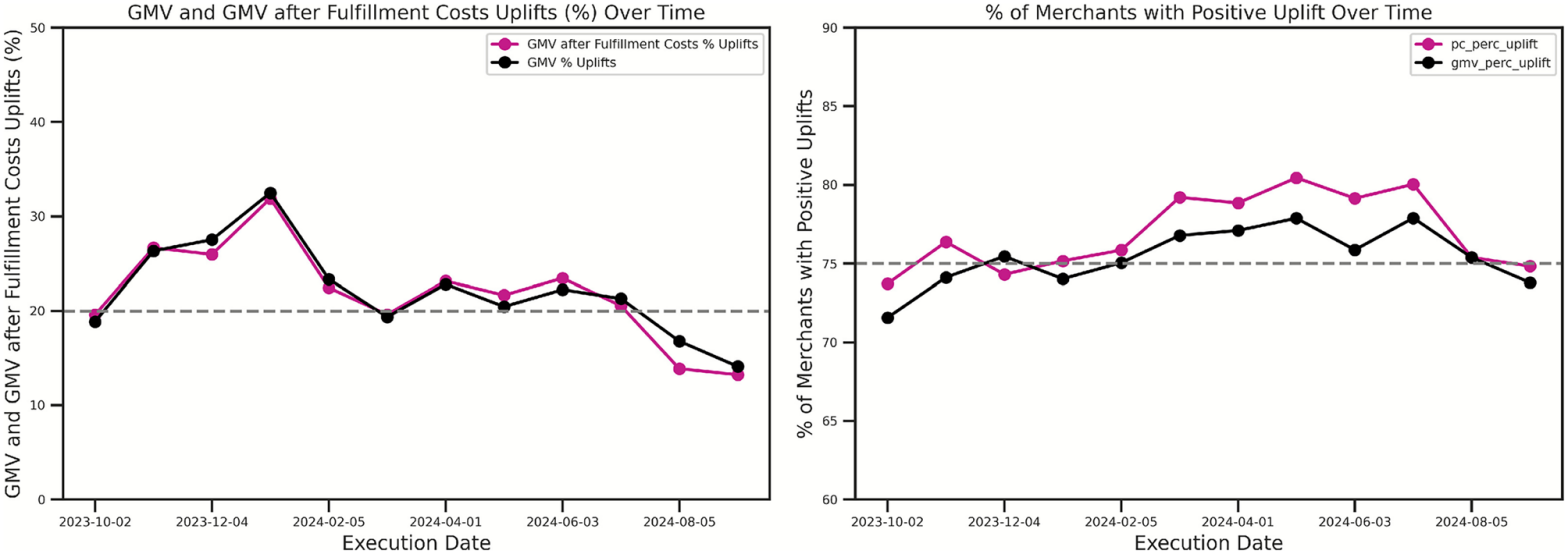
Temporal Stability of GMV and GMV After Fulfillment Costs Uplifts. There is a consistent positive trend in Gross Merchandise Value (GMV) and GMV after fulfillment costs over a 6-week backtest horizon (October 2023–September 2024). A peak in uplifts is shown during January and a trough in September, with $70\%$ to $80\%$ of merchants consistently experiencing positive GMV uplifts throughout the period.

The percentage of merchants with positive GMV and PC uplifts remains stable over time and this is shown in Fig. 4. Over a 6-week backtest horizon, from October 2023 to September 2024, the analysis reveals consistently positive Gross Merchandise Value (GMV) and GMV after fulfillment costs, exhibiting a peak in January and a trough in September. Furthermore, the percentage of merchants experiencing positive GMV uplifts remained robust, ranging between $70\%$ and $80\%$ throughout the period, suggesting a generally effective strategy with potential seasonal influences.

**Fig. 5:**
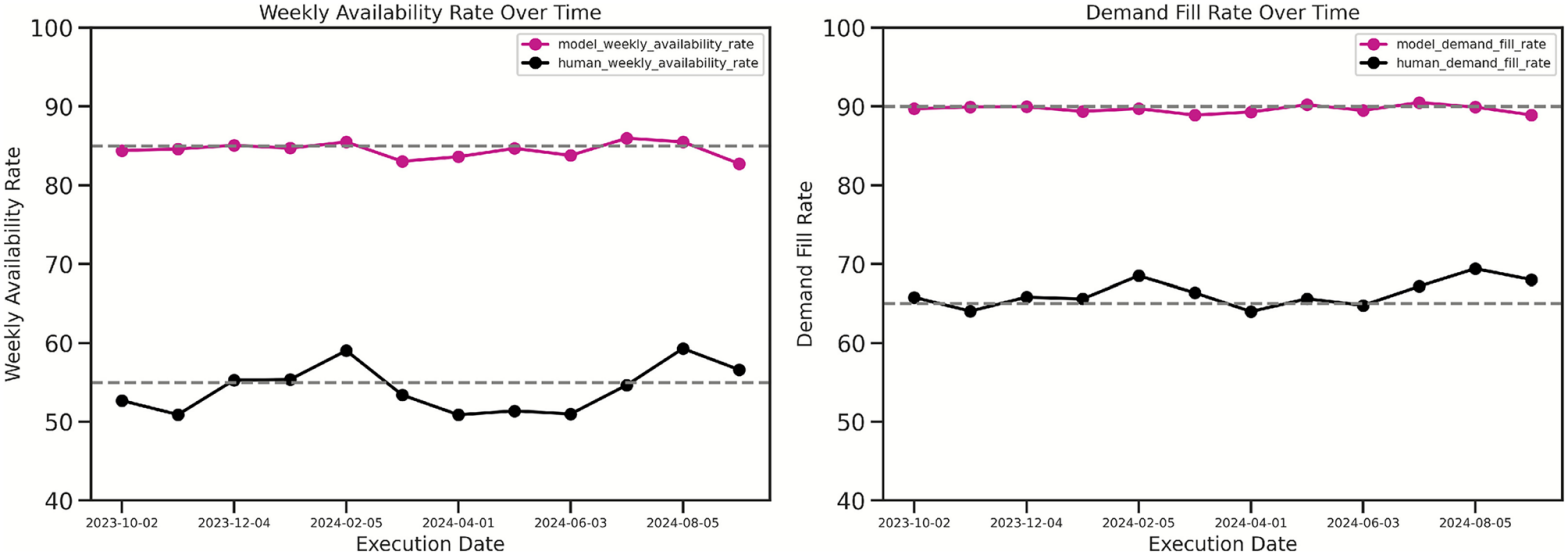
Operational Performance of the Replenishment Recommender Versus Human Benchmarks. This plot illustrates the consistent outperformance of the model over human capabilities in key operational metrics, maintaining a stable weekly availability rate of $85\%$ and a demand fill rate of $90\%$, thereby exceeding human performance in both areas.

The model consistently outperforms human capabilities in terms of operational metrics. Figure 5 demonstrates a higher availability rate and demand fill rate. Specifically, the model maintains a relatively stable weekly availability rate of $\mathbf {85\%}$ and a demand fill rate of $\mathbf {90\%}$, exceeding human performance.

### The efficacy of probabilistic forecasting integration

Our results, particularly the enhanced gross merchandise value and improved service level metrics, underscore the theoretical and practical value of leveraging full demand distributions (via Conformal Prediction quantiles from the LightGBM model) rather than relying solely on deterministic point forecasts. Unlike traditional approaches where forecast errors propagate directly into suboptimal inventory decisions, our method’s ability to sample from the estimated demand distribution within the Monte Carlo simulation allows the optimization engine to proactively account for demand variability. This enables the policy to be robust against unexpected demand fluctuations, minimizing both stock-out costs and overstocking risks, which is crucial in highly uncertain dynamic retail environments.

**Fig. 6:**
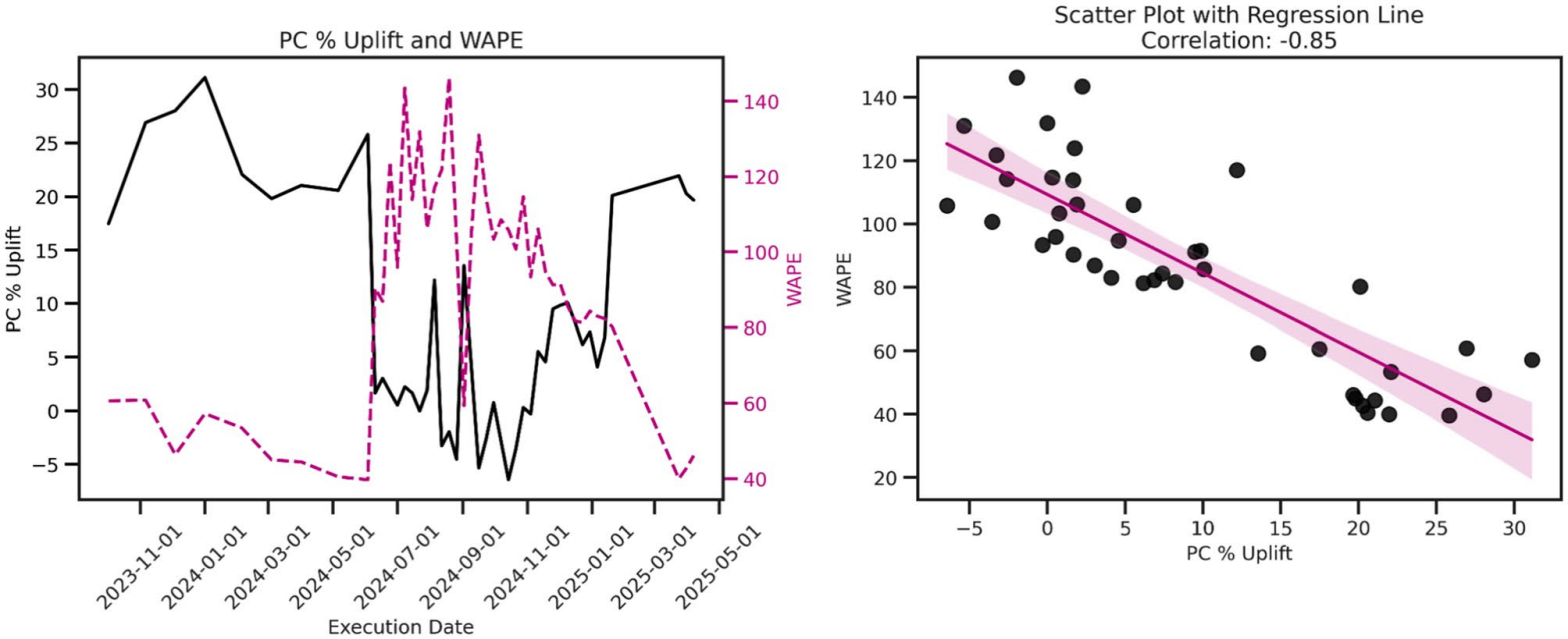
PC % Uplift and WAPE Correlation. We saw a strong negative correlation between PC% Uplift and WAPE, where higher WAPE is associated with low PC uplift.

A key observation from our work concerns the complex relationship between demand forecast accuracy and system performance. We observe a strong negative correlation ($\rho =-0.85$) between demand forecast WAPE and profit contribution percentage uplift (see Fig. 6), indicating that higher WAPE is associated with lower profit contribution. This aligns with intuitive expectations regarding forecast precision and financial outcomes.

**Fig. 7:**
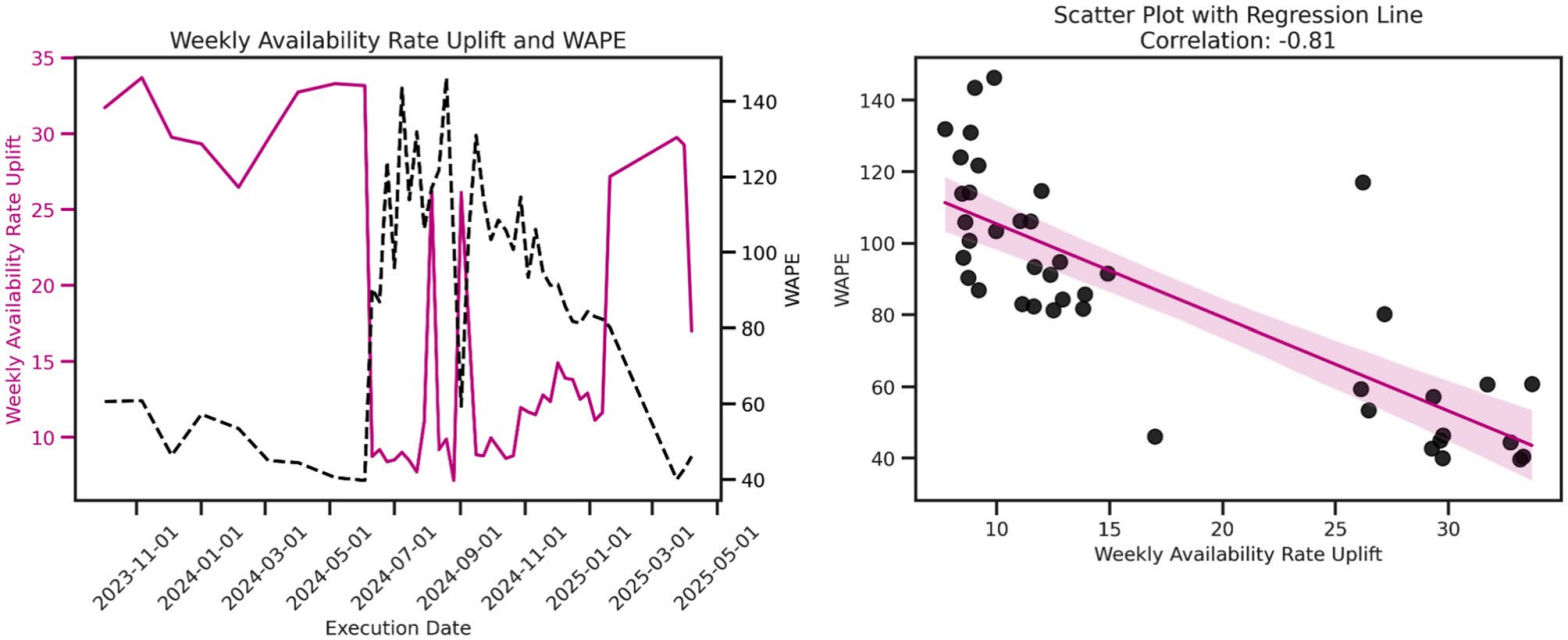
WAPE and Weekly Availability Rate Uplift Correlation. We observe a strong negative correlation between WAPE and availability rate uplift, where higher WAPE is associated with lower uplift in availability.

**Fig. 8:**
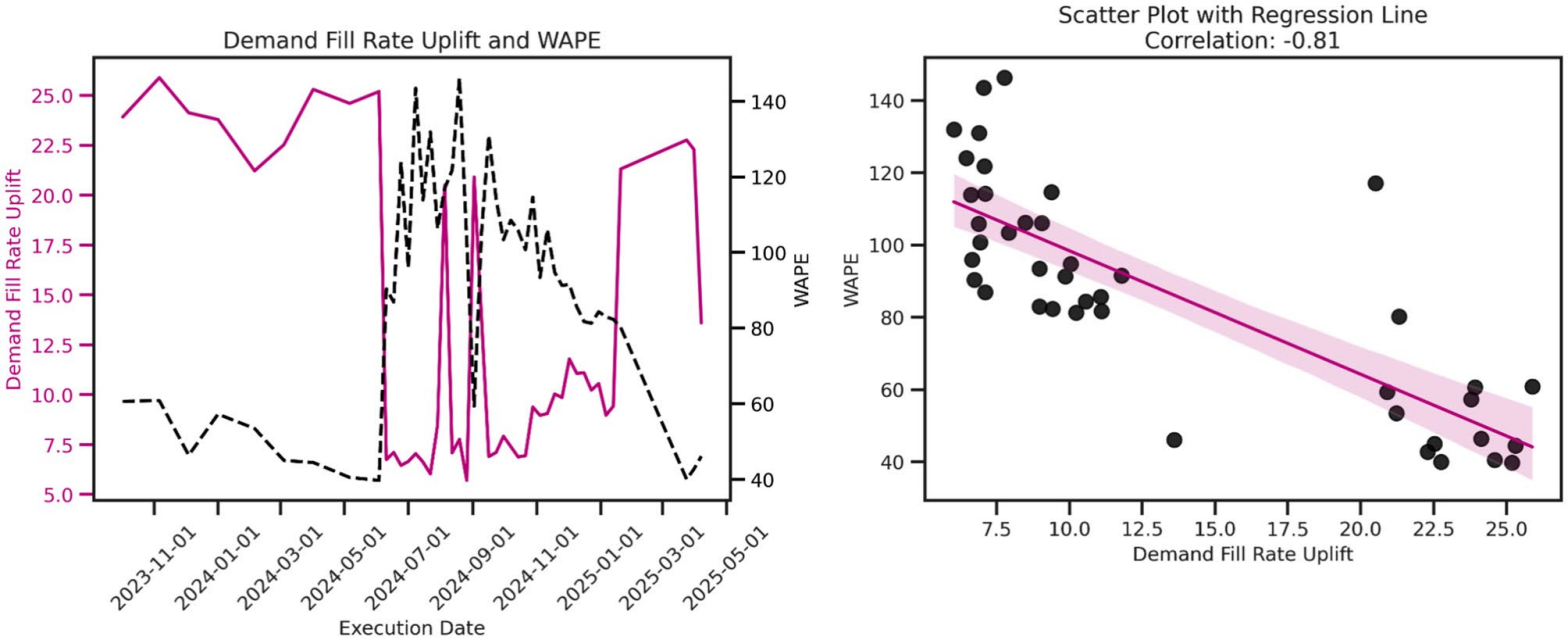
WAPE and Demand Fill Rate Uplift Correlation. We observe a strong negative correlation between WAPE and demand fill rate uplift, where higher WAPE is associated with lower uplift in demand fill rate.

Similarly, when examining operational metrics (see Figs. 7 and 8), our analysis revealed a strong negative correlation ($\rho =-0.81$) between WAPE and service level uplifts (specifically, for both availability rate and demand fill rate). This indicates that lower WAPE (more accurate demand forecasts) generally leads to higher uplifts in these service levels.

However, our findings emphasize that even with higher WAPE, the system can still achieve positive uplifts in availability and demand fill rates, albeit potentially lower than those achieved with highly accurate forecasts.

A plausible explanation for this resilience lies in the stochasticity inherent in our approach. Probabilistic forecasting, coupled with Monte Carlo simulations, effectively captures demand variability. This allows the robust optimization strategy (e.g., using a percentile of the cost distribution) to stabilize results, leading to significant performance enhancements even when individual point forecasts are moderately erroneous. By prioritizing resilience to demand volatility, such a system can ensure high availability and fill rates, with observed ranges consistently above $85\%$ for both metrics, potentially at the expense of optimal profit contribution in scenarios of high WAPE.

### Ablation study

To quantify the contribution of key design choices in the ZEOS Inventory Optimization Tool, we performed an ablation study focusing on two main components of the optimization framework: probabilistic demand forecasting versus deterministic point forecasts, and percentile-based robust objective versus a traditional mean-based objective. Three variants were evaluated as shown in Table 4:

**Table 4:** Ablation Study: Impact of Forecast Type and Objective Function. This table provides the definition and description of the three policy variants used in the study: (i) P-PCTL (point forecast + percentile objective), (ii) Q-MEAN (probabilistic forecast + mean objective), and (iii) Q-PCTL (probabilistic forecast + percentile objective, ours).

Config	Forecast Type	Cost Objective	Description
Q-PCTL (ours)	Probabilistic	Percentile	Full model – stochastic forecast with percentile-based objective (baseline minimizing 75th percentile of cost).
P-PCTL	Point	Percentile	Deterministic forecast with objective minimizing the 75th percentile of cost.
Q-MEAN	Probabilistic	Mean	Probabilistic forecast using demand quantiles but optimizing the mean cost.

All model configurations were rigorously backtested using identical Monte Carlo settings and cost formulations. Performance metrics were evaluated against a human baseline over a 6-week backtest horizon, utilizing a full year of data from October 2023 to September 2024 (12 monthly execution dates). Statistical rigor was maintained by deriving $95\%$ confidence intervals using a merchant-level bootstrap ($B=2000$) and calculating proportions (such as the percentage of merchants with positive GMV uplift) with Wilson score intervals.

**Table 5:** Ablation Study: Impact of Forecast Type and Objective Function. Comparison of (i) P-PCTL (point forecast + percentile objective), (ii) Q-MEAN (probabilistic forecast + mean objective), and (iii) Q-PCTL (probabilistic forecast + percentile objective, ours), using identical data, cost, and simulation settings. All results are reported covering 1 full year, from October 2023 to September 2024, with a 6-week backtest horizon. Metrics include GMV uplift, GMV after fulfillment costs uplift, proportion of merchants with positive GMV uplifts, weighted weekly availability, weighted demand fill rate, and corresponding uplifts versus human benchmarks (pp = percentage points). Confidence intervals ($95 \%$) are obtained via merchant-level bootstrapping and Wilson score methods.

Configuration	GMV Uplift wrt Human	Uplift of GMV After Fulfillment costs wrt Human	% Merchants with Positive GMV Uplifts	Weighted Weekly Availability Rate	Weighted Demand Fill Rate	Weighted Weekly Availability rate uplift wrt Human	Weighted Demand fill rate uplift wrt Human
Q-PCTL (ours)	$22.11\%$ [20.44, 23.92]	$21.95\%$ $[20.03\%, 24.03\%]$	$75.36\%$ [74.34, 76.34]	86.40 [86.18, 86.63]	91.14 [91.00, 91.28]	$+33.63\%$ $[+32.70, +34.59]$ $+21.75\text {pp}$ $[+21.29, +22.19]$	$+23.63\%$ $[+22.99, +24.26]$ $+17.42\text {pp}$ $[+17.05, +17.78]$
P-PCTL	$6.37\%$ $[4.77\%, 8.05\%]$	$5.98\%$ $[4.20\%, 7.88\%]$	$63.20\%$ $[62.03\%, 64.35\%]$	77.76 [77.41, 78.14]	84.95 [84.68, 85.21]	$+18.60\%$ $[+17.74\%, +19.45\%]$ $+12.19\text {pp}$ $[+11.73, +12.67]$	$+13.68\%$ $[+13.12\%, +14.28\%]$ $+10.22\text {pp}$ $[+9.84, +10.59]$
Q-MEAN	$19.02\%$ $[17.53\%, 20.54\%]$	$20.16\%$ $[18.49\%, 22.01\%]$	$69.19\%$ $[68.09\%, 70.28\%]$	81.27 [80.94, 81.59]	87.98 [87.77, 88.19]	$+24.35\%$ $[+23.48\%, +25.24\%]$ $+15.92\text {pp}$ $[+15.43, +16.37]$	$+18.40\%$ $[+17.80\%, +18.99\%]$ $+13.67\text {pp}$ $[+13.30, +14.05]$

The ablation study in Table 5 confirms that both components–probabilistic forecasting and robust objective optimization–contribute meaningfully to overall performance. The shift from P-PCTL (point forecast + percentile objective, showing a GMV uplift of $6.37\%$ and GMV after fulfilment costs uplift of $5.98\%$ compared to the human baseline) to Q-MEAN (probabilistic forecast + mean objective) raises the GMV uplift to $19.02\%$ and the GMV after fulfilment costs uplift to $20.16\%$, validating the theoretical expectation that incorporating uncertainty distributions is crucial. Comparing Q-MEAN to Q-PCTL (our full model) shows a significant numerical improvement in GMV uplift (from $19.02\%$ to $22.11\%$). Furthermore, the uplift in GMV after fulfillment costs for Q-PCTL ($21.95\%$) is also higher than for Q-MEAN ($20.16\%$). The percentile-based criterion provides crucial resilience to stochastic demand shocks, resulting in more consistent and robust outcomes across heterogeneous portfolios.

### Sensitivity analysis

To validate our core design choices, sensitivity analyses were conducted on execution date 2024-09-02, corresponding to the least-uplift date within the year to provide a conservative assessment of robustness. Two critical parameters were systematically evaluated: simulation horizon length and cost percentile objective.

*Simulation-Horizon Sensitivity* confirmed the superiority of our 12-week simulation horizon across all key metrics. The 12-week configuration achieved GMV uplifts of $14.11\%$ and GMV after fulfillment costs uplifts of $13.23\%$, substantially outperforming 8-week ($11.85\%$ GMV, $11.85\%$ after costs) and 6-week ($6.68\%$ GMV, $6.73\%$ after costs) configurations, with the highest merchant success rate ($73.61\%$ experiencing positive GMV uplifts). Notably, the sustained uplift after accounting for fulfillment costs validates the 12-week horizon’s cost-effectiveness and capital efficiency. This extended horizon enables comprehensive lifecycle planning, capturing delayed effects such as product returns and seasonal demand shifts that shorter horizons miss. The exponential cost decay weighting amplifies this advantage by discounting late-period volatility, allowing longer horizons to optimize for both immediate responsiveness and long-term cost stability.

*Cost Percentile Sensitivity* validated our $75^{\text {th}}$ percentile objective as optimal for balancing profitability with operational reliability. While the 90th percentile achieved slightly higher GMV uplifts ($16.06\%$ GMV, $16.98\%$ after costs), the $75^{\text {th}}$ percentile ($14.11\%$ GMV, $13.23\%$ after costs) demonstrated superior service performance with the highest availability uplifts ($+21.08\text {pp}$) and fill rate improvements ($+17.71\text {pp}$), alongside the greatest proportion of merchants experiencing positive outcomes ($73.61\%$). The consistent performance across both GMV and post-fulfillment metrics confirms that financial benefits persist after operational deductions. This threshold approximates Conditional Value-at-Risk (CVaR) principles while maintaining computational stability within our Monte Carlo framework, consistent with industry-standard service levels (75-95%) and empirically observed managerial risk preferences.

Together, these analyses confirm that our 12-week, $75^{\text {th}}$ percentile configuration achieves the optimal balance between financial performance, operational excellence, and robustness under uncertainty. Full sensitivity analysis data and methodology are detailed in the Supplementary Material.

### Backtest performance with uncertainty and informative slices

The model’s backtest performance was rigorously evaluated using paired permutation tests, nonparametric bootstrapping, and aggregate-ratio metrics across the full 12 runs spanning the year (October 2023 to September 2024), merchant, month, and segmented operational levels. Paired tests confirmed statistically significant improvements in operational metrics, showing an availability uplift of $+19.9$ percentage points (pp) and a fill-rate uplift of $+16.3$ pp. Mean per-merchant GMV uplift was also strongly significant at $+34.6\%$. Although the mean per-merchant Profit Contribution (PC) uplift was non-significant ($p_{\text {perm}}=0.4951$) due to high cross-merchant heterogeneity, this outcome is not surprising; it reflects that certain merchants are fundamentally unprofitable or exhibit high cost-to-serve ratios due to inherent inventory challenges, low sales volume leading to statistical instability, or difficult-to-forecast product profiles.

However, the overall aggregate-ratio uplifts–which correctly account for operational scale–were highly stable and significant, confirming robust, system-wide financial gains of approximately $+22\%$ in both PC and GMV over the 12-month period. Analysis by informative slices further demonstrated the robustness of these gains: performance was strongest for high-volume SKU demand categories (Category A: $+22\%$ uplift) and was consistently positive across all merchant scales (ranging from $19\%$ to $33\%$ gains). Furthermore, the model exhibited reliable performance across all seasons (ranging from $20\%$ to $28\%$ uplift), with the strongest gains occurring during the high-demand Winter cycle, confirming its dependable functionality under diverse market conditions. Full data on these analyses are detailed in the Supplementary Material.

### Adaptability of the extended $\left( R, s, Q\right)$ policy

The extension of the classical policy to include $t_0$, $Q_0$, and $t_{\text {limit}}$ proved instrumental in optimizing replenishment strategies for the operational context of large-scale retail. The introduction of $t_0$ and $Q_0$ enables a precisely timed and sized initial replenishment, crucial for optimizing for initial demand capture or aligning with market events, a characteristic particularly salient in seasonal retail due to short product lifecycles and pronounced seasonality. The $t_{\text {limit}}$ parameter facilitates proactive inventory divestment, thereby mitigating costly end-of-season overstock scenarios. This flexibility contrasts with rigid classical policies that might struggle to adapt to the nuanced lifecycle of seasonal articles, highlighting the operational relevance of a tailored decision space. While increasing decision variables expands the search space and allows for greater optimization, it also increases computational complexity. Our use of SHGO, a gradient-free black-box optimizer, mitigated this challenge, demonstrating that practical solutions can be achieved even when analytical gradients are intractable. The literature suggests incorporating learning approaches for sequence optimization may offer future improvements^35–37^.

## Conclusion

### Research contribution and methodology

This study introduced the ZEOS Inventory Optimization Tool, a novel system designed to bridge the methodological gap between probabilistic forecasting and prescriptive policy optimization in dynamic e-commerce.

The ZEOS Inventory Optimization Tool establishes three core methodological innovations:

1. **Probabilistic Optimization Architecture:** It institutes an end-to-end architecture where demand forecast uncertainty (from a LightGBM/Conformal Prediction model) is propagated via Monte Carlo–based simulations, allowing the system to anticipate demand variability rather than react to point forecasts. This approach explicitly leverages methods for rigorous uncertainty quantification^17^.
2. **Extended **$\left( R, s, Q\right)$ **Formulation:** It significantly extends the classical $\left( R, s, Q\right)$ policy (Jansen, 1996) by jointly optimizing five decision variables–including initial replenishment time ($t_0$), initial quantity ($Q_0$), and a termination time ($t_{\text {limit}}$)–to capture the entire product lifecycle from launch to withdrawal. The incorporation of fulfillment-time decision-making aligns with complex modern online retail environments^21^.
3. **Risk-Aware Objective:** The model employs a percentile-based objective function, minimizing the $75^{\text {th}}$ percentile of total cost, which is analogous to a Conditional Value-at-Risk (CVaR) criterion. This approach mitigates tail-risk scenarios and explicitly balances profitability with service-level stability, informed by robust optimization theory^31^.

The practical deployment of this approach requires integrating the Probabilistic Forecasting Engine, a Discrete-Event Simulation (DES) module, and a Black-Box Optimization Engine (e.g., SHGO).

### Empirical findings and resilience

The system delivers substantial and statistically robust performance improvements validated across a comprehensive 12-month backtest (October 2023 – September 2024) covering over 800 merchants:

- **Financial Uplift:** Based on our evaluation methodology, portfolio-level performance relative to human benchmarks showed gains of approximately $\mathbf {22\%}$ in GMV and GMV after fulfilment costs uplifts, with median uplifts between $19.45\%$ and $22.11\%$. The sustained uplift after accounting for fulfillment costs validates the strategy’s cost-effectiveness and capital efficiency.
- **Operational Excellence:** The model consistently outperformed human decisions, maintaining weekly availability above $85\%$ and fill rates exceeding $90\%$, corresponding to average uplifts of $+19.9$ percentage points (pp) and $+16.3$ pp, respectively.
- **Validation of Core Design:** Ablation analysis (Table 5) confirms the strength of the full model (probabilistic-percentile), achieving a superior GMV uplift of $22.11\%$ and GMV after fulfilment costs uplift of $21.95\%$ compare to the probabilistic-mean configuration, which achieved a $19.02\%$ GMV uplift and $20.16\%$ GMV after fulfilment costs uplift, and the point-percentile configuration, which achieved a $6.37\%$ GMV uplift and $5.98\%$ GMV after fulfilment costs uplift.
- **Resilience under Noise:** The backtest revealed a strong negative correlation ($\rho \approx -0.85$) between forecast error (WAPE) and financial performance. Crucially, the system’s stochastic and robust design successfully stabilized outcomes, consistently producing positive KPI gains even under high forecast uncertainty. Specifically, the model can still achieve positive uplifts in availability and demand fill rates (observed ranges consistently above $85\%$ for both metrics) even with higher WAPE, prioritizing resilience and service levels at the expense of potentially optimal profit contribution.

### Practical adoption of the method

To operationalize this approach, businesses must integrate three core components: probabilistic forecasting, stochastic simulation, and black-box optimization.

- **Probabilistic Forecasting Engine** – A model such as LightGBM should be trained on rich temporal, product, and merchant-level features to produce predictive distributions (quantiles or intervals) rather than single-point forecasts, enabling uncertainty-aware planning. This acknowledges recent advances in forecasting for complex retail contexts^14^.
- **Discrete-Event Simulation (DES)** – The DES module replicates supply chain dynamics under uncertainty, using Monte Carlo sampling to estimate a cost distribution $C(\theta )$ for each replenishment policy $\theta$.
- **Black-Box Optimization Engine** – A global optimizer (e.g., SHGO) searches the parameter space $\{t_0, Q_0, s, Q, t_{\text {limit}}\}$ to minimize a robust percentile (e.g., $75^{\text {th}}$) of the simulated cost distribution, identifying policies that perform reliably across stochastic demand scenarios.

Together, these components transform inventory control from a reactive, rule-based process into a proactive, data-driven system that explicitly models uncertainty, enhancing both profitability and service stability.

### Limitations and future work

While the ZEOS Inventory Optimization Tool provides a scalable, data-driven solution, its current tailoring to Zalando Fulfillment Solutions (ZFS) restricts immediate cross-industry generalizability, necessitating broader validation. Additionally, the model assumes high accuracy in input data (e.g., demand forecasts, return estimations, and inbound reports), which may not consistently reflect real-world noisy or incomplete data, potentially impacting robustness. First, this assumption may introduce a systemic bias in our evaluation, potentially overstating performance in an ideal environment. Second, it suggests that the model’s robustness and performance may be highly sensitive to differences in data noise and incompleteness levels across alternative organizational settings, posing a challenge for implementation in other contexts.

Future work will focus on expanding applicability to diverse industries, encompassing cross-business model optimization beyond ZFS. Methodologically, we plan to explore more sophisticated policies not yet fully addressed by the current framework, such as integrating dynamic programming approaches or Reinforcement Learning (RL) for sequence optimization, which may offer improvements over the current one-shot policy optimization solved by the SHGO algorithm^35–37^. Further research will also include the exploration of how incorporating lead time and return variance affects overall performance, integrating external events (e.g., major promotions), enhancing risk management for end-of-season overstock, and optimizing policies for low-performing articles. These enhancements aim to further strengthen the tool’s practical utility and adaptability.

## Supplementary Information

Supplementary Information.

## Data Availability

The datasets used and/or analysed during the current study available from the corresponding author on reasonable request.
