# Correction: A practical approach to replenishment optimization with extended (*R*, *s*, *Q*) policy and probabilistic models

**DOI:** 10.1038/s41598-026-37090-0

**Published:** 2026-01-30

**Authors:** Alva Presbitero, Andreas Syrén, Hagop Dippel, Lalita Awasthi, Shikhar Dev, Zhe Nie

**Affiliations:** Zalando, 10243 Berlin, Germany

Correction to: *Scientific Reports* 10.1038/s41598-025-32537-2, published online 19 December 2025

In the original version of the Article, Figure 2 was a duplication of Figure 1.

The original Article has been corrected.

