# Supplementary material for: A practical approach to replenishment optimization with extended (R, s, Q) policy and probabilistic models
Source: Sci Rep. 2025 Dec 19;15:44225. doi: 10.1038/s41598-025-32537-2 (PMC12722329; doi:10.1038/s41598-025-32537-2)
Supplement: Supplementary file 1 — Supplementary Information. [file 41598_2025_32537_MOESM1_ESM.docx]

Supplementary Material

# 1. Decision Variables in the Replenishment Policy

We parameterize the replenishment policy using the following decision variables:

$t_{0}$ **:** initial time of replenishment.

$Q_{0}$ **:** quantity ordered in the initial replenishment

$s$ **:** reorder threshold at which additional replenishment is triggered

$Q$ **:** quantity ordered in subsequent replenishments

$t_{limit}$ **​:** termination point for the policy's applicability

In this formulation, the review frequency ($R$) is treated as a fixed parameter configured externally rather than as a decision variable, allowing for more adaptable policy designs. The only two decision variables that are actually shown to the user are the next replenishment variables ${(t}_{0},Q_{0})$. Figure 1 summarises the replenishment policy as parametrized in the ZEOS Inventory Optimization Tool.


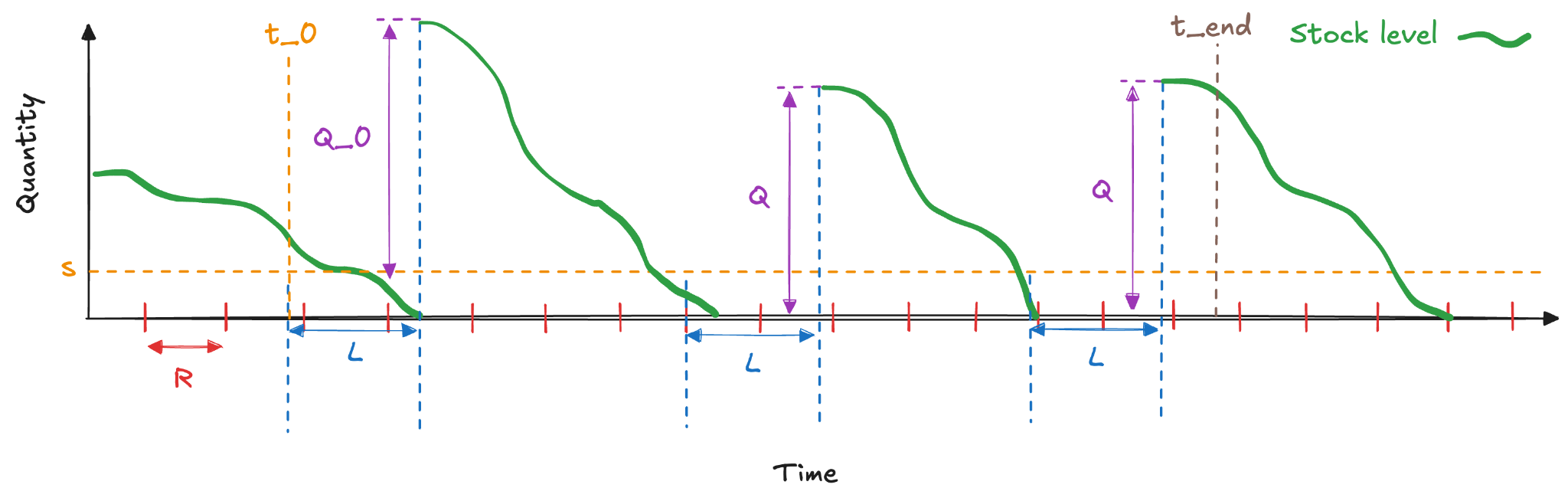


Figure 1. Illustration of a replenishment policy as parameterized in the ZEOS Inventory Optimization Tool. $R$ is the review frequency, L is the replenishment lead time, $Q_{0}$ is the initial replenishment quantity while $Q$ are subsequent replenishment quantities.

# 2. Demand Forecast Model

1. **Sale extrapolation**

We define demand as in-stock sales. Accordingly, observed sales are classified into in-stock and stock-out sales. Both sales and demand are considered at the configuration SKU (cSKU, color) level, where "in-stock" refers to all individual SKUs (sSKU, size) of a given color being available. The sale extrapolation process is a preprocessing step that converts sales data into demand data, facilitating direct demand modeling and leveraging a larger pool of sales data.

To extrapolate cSKU sales, we employ sales and stock availability data at the size level. For each cSKU, in-stock sales at the size level (represented in purple in the middle plot) serve as anchor points. Using the demand distribution at the size level (depicted in the left plot), the demand for missing sizes is calculated (shown in the right plot).


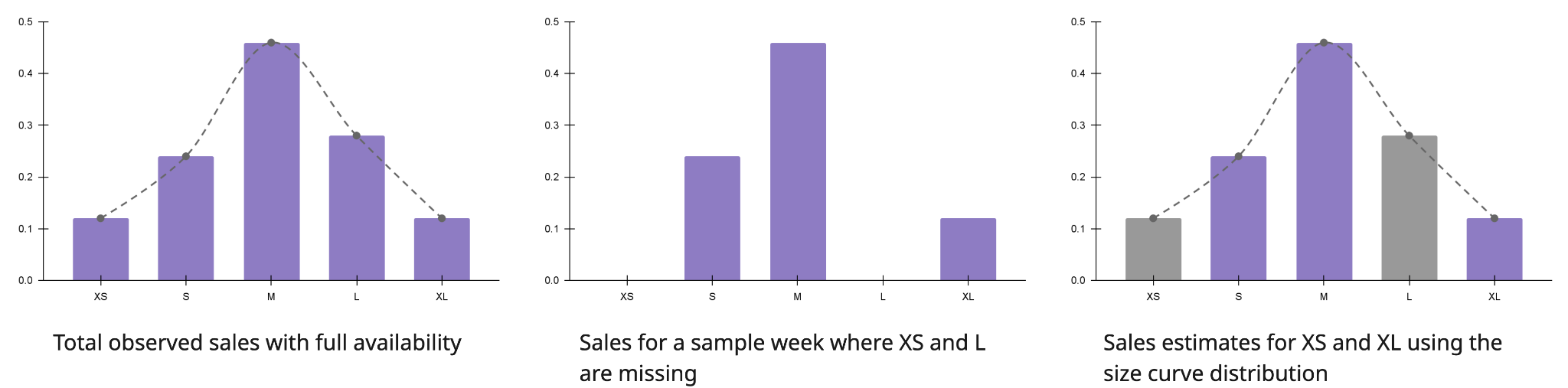


Fig. 2 Illustration of sale extrapolation with size distribution

The current sales extrapolation methodology is evaluated by substituting the in-stock size with a stock-out size and comparing the resulting extrapolated demand against the actual demand. The method yields a **Weighted Average Percentage Error (WAPE)** of 6%, though it is important to note that sample selection bias may also contribute to this result.

Some key challenges for the current sales extrapolation method are below:

1. **Ineffectiveness for Single-Size SKUs:** The method proves ineffective for SKUs that consist of a single size, as it does not account for variations in size distribution.
2. **Limitations During Full Stock-Outs:** The methodology fails when all sizes of a SKU are out of stock, as it lacks a reliable anchor point for extrapolation.
3. **Assumption of Static Demand Size Distribution:** The model assumes that the demand size distribution is static, which can lead to inaccuracies, particularly for SKUs with limited historical data.
4. **Lack of Generalization Across SKUs:** The method does not generalize well across different SKUs, limiting its applicability to a wider range of products.
5. **Exclusion of Cannibalization Effects:** The methodology does not consider cannibalization, potentially resulting in overestimations of aggregate demand. This limitation can lead to inaccuracies when forecasting total demand at the SKU level.
6. **Demand Forecasting**

The demand is extrapolated from sale by available articles. So the demand is assumed accurate and used as labels for demand modeling, framed as a time series regression problem. The dataset is organized into time series, each representing one SKU over time, with each data point as a vector of features for a specific week (e.g.,sales, availability, price, time, article static information; see Feature Engineering). Demand forecasting is conducted at the SKU and weekly level.

To improve the quality of the dataset and avoid bias introduced by sparse time series, a **dense representation** of the data is used. A dense representation in the context of this time series model refers to filling in missing data points, such as those during stock-out weeks, using imputation methods to ensure the time series is continuous, which prevents bias and improves the robustness of demand forecasts. Missing data points, including stock-out weeks, are filled using appropriate imputation methods, ensuring that the time series remains continuous. This approach allows the model to better capture temporal patterns without being influenced by gaps in the data. By representing the time series in a dense format, we ensure that the training data contains all necessary anchor points, improving the quality and robustness of the demand forecasts.

The demand forecasting is applied to replenishment recommendations, with results formatted for compatibility with the recommendation engine’s sampling and stock simulation. The result schema includes indexes such as config_sku, merchant_id, time index, and quantiles (q1, q5, q10, ..., q95, q99). These quantiles are derived from confidence intervals generated using conformal prediction, providing a probabilistic representation of demand distributions for downstream simulations.

The forecasting model operates at the configuration SKU and week level, with a 12-week horizon. The inference scope is restricted to "active" SKUs—defined as those with positive stock or sales in the past 18 weeks. This scope includes 2 million simple SKUs and about 70,000 configuration SKUs (depending on execution dates).

Feature engineering is performed before training, linking various feature types (e.g., article info, time, holidays, stock, prices, sales) to generate the training dataset.

# 3. Model Details & Engineering:

The **LightGBM model** provided by **MLForecast from Nixtla** was selected for time series forecasting. **MLForecast** is a high-performance Python library designed for time series forecasting using gradient-boosted decision trees like LightGBM. It simplifies the process of training, evaluating, and deploying forecasting models by integrating efficient handling of time series features, enabling scalability to large datasets. LightGBM is particularly well-suited for structured data forecasting tasks due to its ability to handle large-scale datasets and capture complex interactions between features with high computational efficiency.

In this project, MLForecast with LightGBM was leveraged to train a regression-based model for demand forecasting. The model was designed to predict demand at the configuration SKU level over a 12-week horizon. Instead of directly optimizing quantiles through a quantile loss function, Conformal Prediction was used to compute confidence intervals, which were then transformed into quantiles (q2.5, q5, q10, ..., q95, q97.5). This approach ensures that the derived quantiles are probabilistically valid and reflect the uncertainty of the forecasts accurately.

Conformal Prediction is a statistical framework that provides prediction intervals with a predefined confidence level, offering robust uncertainty quantification. This method ensures that the prediction intervals are consistent with the desired coverage, regardless of data distribution or noise. By transforming these intervals into quantiles, we can capture demand distributions while retaining the rigor and reliability of Conformal Prediction.

The MLForecast library provides tools for automatically generating lag-based features, rolling windows, and other time-based transformations to enhance model performance. This allowed us to capture temporal patterns and trends in the time series data effectively.

A post-processing step is implemented to incorporate business insights and decompose demand forecasts from the configuration SKU level to the simple SKU level. This decomposition is based on the size distribution, ensuring that the forecasts reflect the specific demand patterns at the individual SKU level.

The evaluation process serves two primary purposes:

1. **Evaluation of the Machine Learning Method:** This involves comparing the performance of the machine learning model with a naive method, which mimics human prediction performance.
2. **Evaluation of the Trained Model:** This focuses on assessing the accuracy and robustness of the trained model's predictions.

**Return Lead Time Model**

The returns model is an integral component of the broader inventory optimization framework, addressing the uncertainty and temporal dynamics of customer returns. By accurately forecasting return flows, the model informs inventory management decisions, ensuring alignment between stock availability and demand. It operates as a probabilistic forecasting mechanism, embedded within the inventory optimization tool to provide insights into the timing and magnitude of returns. Its core formulation is as follows:

${Returns}_{t}=\alpha_{t-1}{Outbound}_{t-1}+\alpha_{t-2}{Outbound}_{t-2}+...+\alpha_{t-T}{Outbound}_{t-T}$

Here, $\alpha_{t}$ denotes the probability of a return from outbound transactions at time $t$. The weighted contributions of lagged outbound data account for return delays, varying customer behaviour, and logistical factors. The model is implemented at multiple levels of granularity, such as overall returns, commodity-specific returns, and configuration-level returns. The configuration-level implementation significantly outperforms broader aggregates, reducing Weighted Absolute Percentage Error (WAPE) and bias by approximately 10 percentage points compared to baseline models. This precise modeling of returns enhances the inventory optimization tool’s capability to handle complex, dynamic systems, contributing to improved operational efficiency and profitability.

The returns sub-model contributes to the inventory optimization tool in the following ways:

1. **Demand Adjustments:** By forecasting returns, the tool can dynamically adjust net demand, reducing the risk of overstocking or understocking.
2. **Lead-Time Variability:** Incorporating return lead-time distributions allows the model to anticipate delayed inventory inflows, ensuring more accurate inventory allocation.
3. **Cost Optimization:** By aligning return forecasts with outbound stock management, the tool minimizes costs associated with surplus inventory and reprocessing returned items.

**Replenishment Lead Time Model**

To model the variability in lead times announced by merchants, we implemented a sampling method based on a gamma distribution. This approach accounts for the inherent uncertainty and variability in lead times while ensuring that the sampled values remain within reasonable bounds. The process is outlined as follows:

The expected lead time, denoted as EEE, is provided by the merchant. This value represents the merchant's forecast for the lead time required to fulfill an order, typically based on historical data and operational constraints.

To capture the variability in lead time, we utilize a gamma distribution with the following parameters:

- **Shape Parameter (**$k$**)**: The shape parameter is set to $2E$. This choice ensures that the distribution is centered around the expected lead time while allowing for variability. The factor of 2 allows the distribution to be more spread out, reflecting the natural uncertainty in lead time predictions.
- **Scale Parameter (**$\theta$**)**: The scale parameter is fixed at 0.4. This value controls the spread of the distribution and is chosen based on empirical data, balancing the range of possible lead times with the expected lead time.

A sample lead time $L$ is drawn from the gamma distribution with the specified shape parameter $k$ and scale parameter $\theta$. The sampling process can be mathematically expressed as follows:

$L_{sample}\sim Gamma(k=2E, \theta=0.4)$

After the sample is drawn, the value of $L_{sample}$​ is rounded to the nearest integer to represent whole days, ensuring that the lead time is expressed in discrete units of time.

To ensure that the sampled lead time remains within realistic and operationally feasible limits, two clamping operations are applied:

- **Minimum Clamp**: The sampled lead time is clamped to a minimum value of 1 day. This prevents the lead time from being negative or zero, which would be unrealistic in the context of order fulfillment.
- **Maximum Clamp**: The sampled lead time is also clamped to a maximum value of $2E$, ensuring that the lead time does not exceed twice the expected value. This upper bound prevents the model from generating excessively large lead times that are outside the typical range of merchant operations.

Finally, the clamped and rounded sampled lead time is converted to an integer type for consistency and to ensure compatibility with other components of the forecasting system.

This methodology provides a robust way to simulate lead time variability, taking into account both the expected lead time and inherent uncertainty. By using a gamma distribution with appropriate parameters, we are able to model a realistic range of lead times that reflect the variability observed in historical data.

# 4. Table of Abbreviations

Table 1 provides a comprehensive reference for all the key acronyms and specialized terms used throughout this supplementary material, including their full names and brief descriptions to ensure clarity and consistency.

| **Abbreviation** | **Full Name** | **Description** |
| --- | --- | --- |
| CVaR | Conditional Value at Risk | A measure of tail risk used in operations research; the 75th percentile objective is a tractable approximation of this. |
| DES | Discrete Event Simulation | The core method for evaluating policy costs under uncertainty. |
| GMV | Gross Merchandise Value | Key business performance indicator. |
| KPI | Key Performance Indicator | The practical business metrics (e.g., GMV, Availability, Fill Rate) used to evaluate the |
| LightGBM | Light Gradient Boosting Machine | The machine learning model used for probabilistic forecasting. |
| PC | Profit Contribution | The primary objective function component being maximized. |
| RsQ | Review, Reorder Point, Quantity | The classical inventory control policy with three decision variables: Review, Reorder Point, Quantity |
| SHGO | Simplicial Homology Global Optimization | The gradient-free optimizer used to solve the model. |
| SKU | Stock Keeping Unit | The fundamental inventory tracking unit, in our case a fashion article. |
| WAPE | Weighted Absolute Percentage Error | Forecasting accuracy metric. |
| ZEOS | Zalando Brand Name | Zalando B2B service |
| ZFS | Zalando Fulfillment Solutions | The internal division/platform context for the tool's deployment. |

Table 1: Table of Abbreviations

# 5. Baseline Comparators and Evaluation Framework

**Evaluation Design and Optimization Parity.** To ensure comparability, all algorithmic approaches share identical experimental conditions:
 (i) the same probabilistic demand forecasts,
 (ii) uniform cost-parameter specifications,
 (iii) identical lead-time-uncertainty distributions, and
 (iv) consistent operational constraint sets.

Parameter optimization across all baselines employs the Simplicial Homology Global Optimization (SHGO) algorithm with 500 Monte Carlo simulations, ensuring each policy attains its theoretical performance potential under the same informational environment. Consequently, observed performance differences reflect intrinsic algorithmic efficacy rather than optimization artifacts or information asymmetry.

| **Policy** | **Review Type** | **Decision Rule** | **Parameters** | **Key Characteristics** | **Theoretical Foundation** |
| --- | --- | --- | --- | --- | --- |
| **Our Enhanced R(s,Q)** | Periodic (R periods) | Order Q when inventory ≤ s | s, Q, t₀, Q₀, $t_{limit}$ (R fixed) | Parameters are data-driven (ML). Balances multiple objectives (holding, stockouts, transaction costs). Incorporates demand uncertainty and business constraints. Strategic timing and kickstart advantages. | Operations Research + Machine Learning |
| **Human Baseline** | Variable | Expert judgment | Merchant-defined | Incorporates contextual market knowledge and qualitative factors; flexible adaptation to anomalies. | Human Expertise |
| **Tuned (s,S)** | Continuous | Order up to S when inventory position IP≤s | s, S, $t_{limit}$ | Immediate responsiveness; monitors IP constantly; orders exact difference to reach S. No strategic timing/kickstart advantage - starts week 0. | Classical Inventory Theory |
| **Base-Stock** | Periodic (R periods) | Always order up to target level S at every review. (Order Q=S−IP_t) | S, $t_{limit}$ (R fixed) | Orders placed every review period regardless of trigger; simple rule. No strategic timing/kickstart advantage - starts week 0. | Classical Periodic Review Policy |
| **Myopic Newsvendor** | One-time (Static) | Single order Q∗ at week 0 only | Q∗, $t_{limit}$ | Single-period optimization applied once at the beginning; no subsequent reordering. No strategic timing/kickstart advantage - pure classical approach. | Classical Newsvendor Model |

**Table 2.**  **Inventory Policy Characteristics and Implementation Framework for Machine Learning vs. Classical Baseline Comparison.** Overview of the five inventory policies evaluated in our comparative study. The ML-enhanced R(s,Q) policy leverages machine learning for parameter optimization while respecting business constraints, compared against three classical inventory theory baselines and human expert judgment. All algorithmic approaches utilize identical demand forecasts, cost structures, and lead time distributions, with parameter optimization performed using the same SHGO algorithm and stochastic simulation framework. This design ensures that performance differences reflect algorithmic capabilities rather than informational advantages, enabling rigorous assessment of machine learning value in inventory management.

# 6. Backtest Performance with Uncertainty, Paired Tests, and Informative Slices

This section evaluates the model’s backtest performance using statistically rigorous methods to quantify both central tendencies and uncertainty across multiple aggregation levels. The analysis integrates paired permutation tests, nonparametric bootstrapping, and aggregate-ratio metrics to measure relative improvement in Profit Contribution (PC), Gross Merchandise Value (GMV), and service-level indicators such as availability and fill rate. Results are presented across three complementary perspectives: (1) paired comparisons at merchant and month level, (2) aggregate-ratio estimates summarizing total impact, and (3) informative slices capturing variation by SKU demand category, merchant scale, and seasonality. This multifaceted evaluation enables a robust understanding of both the magnitude and stability of performance uplifts under different market conditions.

**Paired tests (merchant- and month-level)**

| **Metric (unit)** | **Mean** | **95% CI** | **p_perm** | **N** |
| --- | --- | --- | --- | --- |
| PC uplift % (per-merchant mean) | −46.527% | [−164.487, 40.415] | 0.4951 | 818 |
| GMV uplift % (per-merchant mean) | 34.620% | [31.416, 38.045] | 0.0000 | 803 |
| Availability uplift (pp) | 19.918% | [18.787, 21.076] | 0.0000 | 811 |
| Fill-rate uplift (pp) | 16.306% | [15.477, 17.200] | 0.0000 | 811 |

| **Metric (unit)** | **Mean** | **95% CI** | **p_perm** | **Months used** |
| --- | --- | --- | --- | --- |
| Monthly PC uplift % (aggregate ratio per month) | 21.816% | [19.069%, 24.480%] | 0.0006 | 2023-10 to 2024-09 (12 months) |
| Monthly GMV uplift % (aggregate ratio per month) | 22.118% | [19.527%, 24.732%] | 0.0005 | 2023-10 to 2024-09 (12 months) |

**Table 3: Paired Effects Across Merchants and Months.** Paired tests are used to compare the model against human decisions. Per-merchant uplifts are presented as mean percentage differences, while month-level results are reported as aggregate ratios, which calculate the difference between the model's total results and the human baseline's total results, expressed as a percentage of the human baseline's total. "pp" stands for percentage points. The results indicate statistically significant improvements in operational metrics (availability, fill-rate) and Gross Merchandise Value (GMV), but also show substantial variability in Profit Contribution (PC) uplift across different merchants.

The paired tests confirm statistically significant improvements in operational metrics ($p_{perm}$=0.0000), increasing availability by +19.9 pp and fill rate by +16.3 pp. Mean per-merchant GMV uplift is also strongly significant (+34.6%, $p_{perm}$=0.0000), demonstrating a widespread benefit across the portfolio. Conversely, the per-merchant mean PC uplift is non-significant (-46.527%, $p_{perm}$=0.4951), primarily due to substantial cross-merchant heterogeneity and large variance (CI span of ~ 200%). However, when aggregated over the 12-month period, both monthly PC and GMV uplifts are highly stable and significant (~22% uplift, $p_{perm}$ = 0.0006), confirming that the model delivers substantial aggregate profitability gains, particularly when weighted by merchant scale.

**Overall aggregate-ratio uplifts**

| **Metric** | **Point estimate** | **95% CI** |
| --- | --- | --- |
| PC uplift (aggregate) | 21.930% | [18.836%, 27.564%] |
| GMV uplift (aggregate) | 22.098% | [19.574%, 27.226%] |

Table 4: Table 4: Overall Aggregate-Ratio Uplifts. These uplifts are calculated over all merchants, weighting by transaction volume, and represent the difference between the model's total results and the human baseline's total results, expressed as a percentage of the human baseline's total. Ninety-five percent confidence intervals were derived from 2,000 bootstrap resamples at the merchant level.

The overall aggregate-ratio results, weighting by operational scale, confirm system-wide uplifts of approximately +22% in both PC and GMV. The confidence intervals are notably tight (e.g., GMV CI: [19.574%, 27.226%]), reinforcing the statistical reliability and practical significance of the model's performance for large-scale operations. This provides robust evidence that, despite the heterogeneous per-merchant mean PC, the system successfully maximizes profitability when accounting for business volume.

**Informative slices by SKU demand category (A–D)**

(A = top ≈ 70% GMV; B = next 20%; C = next 5%; D = last 5%)

| **Slice** | **PC uplift %** | **95% CI (PC)** | **GMV uplift %** | **95% CI (GMV)** | **N** |
| --- | --- | --- | --- | --- | --- |
| A (70% GMV) | 22.007 | [18.802, 28.108] | 22.160 | [19.531, 27.249] | 702 |
| B (70%-90% GMV) | 6.001 | [1.623, 28.575] | 7.886 | [3.408, 25.521] | 67 |
| C (90%-95% GMV) | 46.563 | [−7.984, 118.680] | 41.293 | [−6.168, 102.449] | 8 |
| D (95%-100% GMV) | 11.802 | [−5.377, 35.094] | 15.388 | [−2.446, 35.864] | 57 |

Table 5: SKU Demand Category Uplifts. Aggregate PC and GMV uplifts for SKU categories defined by cumulative GMV contribution (A = top ~ 70% GMV; B = next 20%; C = next 5%; D = last 5%). Uplifts are aggregate ratios per slice; confidence intervals are derived from merchant-level bootstrapping.

The model performs best for Category A SKUs, which drive the majority of GMV, achieving +22% gains in both PC and GMV. These items represent the most commercially relevant segment, indicating that the model effectively optimizes high-volume inventory. Categories B and D show smaller but positive uplifts, while Category C exhibits wide uncertainty due to small sample size. Overall, the recommender demonstrates reliable uplift where business impact is most concentrated.

**Informative slices by merchant ABCD (GMV share)**

| **Slice** | **PC uplift %** | **95% CI (PC)** | **GMV uplift %** | **95% CI (GMV)** | **N** |
| --- | --- | --- | --- | --- | --- |
| A (70% GMV) | 18.527 | [14.485, 25.387] | 18.979 | [15.936, 24.766] | 20 |
| B (70%–90% GMV) | 32.580 | [29.695, 35.695] | 32.674 | [29.858, 35.560] | 68 |
| C (90%–95% GMV) | 24.498 | [21.012, 28.424] | 23.911 | [20.453, 27.868] | 62 |
| D (95%–100% GMV) | 28.028 | [25.470, 30.793] | 26.404 | [23.968, 29.072] | 684 |

Table 6: Merchant Scale Uplifts. Aggregate PC and GMV uplifts for merchant segments defined by cumulative GMV contribution (A = top ~ 70% GMV; B = next 20%; C = next 5%; D = last 5%). Uplifts are aggregate ratios per slice; confidence intervals are derived from merchant-level bootstrapping.

Across merchant tiers, all groups show significant positive uplifts. Mid-tier and smaller merchants (segments B and D) achieve the largest improvements (~26–33%), while large merchants (A) gain ~19%. The close correspondence between PC and GMV uplifts indicates that profit and sales benefits scale together, validating the model’s robustness across diverse merchant profiles and business volumes.

**Informative slices by season (merchant–month)**

| **Season** | **PC uplift %** | **95% CI (PC)** | **GMV uplift %** | **95% CI (GMV)** | **N** |
| --- | --- | --- | --- | --- | --- |
| Autumn | 20.868 | [16.056, 28.819] | 20.562 | [16.538, 28.108] | 761 |
| Spring | 21.372 | [18.296, 27.094] | 20.715 | [18.118, 26.117] | 643 |
| Summer | 19.943 | [16.570, 25.647] | 20.433 | [17.881, 25.086] | 654 |
| Winter | 26.808 | [24.142, 30.824] | 27.777 | [25.405, 31.176] | 691 |

**Table 7: Seasonal Uplifts.** Aggregate PC and GMV uplifts for seasons based on merchant–month execution dates (Winter = Dec–Feb, Spring = Mar–May, Summer = Jun–Aug, Autumn = Sep–Nov). Uplifts are aggregate ratios per slice; confidence intervals are derived from merchant-level bootstrapping.

The model exhibits consistent positive uplifts across all seasons, with PC and GMV improvements between 20% and 28%. The strongest gains occur during Winter (~27% uplift), aligning with high-demand retail cycles, suggesting that the model adapts well to periods of increased sales volatility. Seasonal consistency further confirms that performance gains are robust to external market variation and temporal dynamics.

# 7. Sensitivity analysis

**Simulation-Horizon Sensitivity (6, 8, and 12 weeks)**

The differences in results across the 6, 8, and 12-week simulation horizons stem from the trade-off between the model's planning foresight and the temporal weighting of costs. The simulation horizon directly dictates the model's forward-looking capacity. Conversely, the exponential decay applied to cost components biases the optimization process, causing it to prioritize and favor near-term outcomes. The use of exec_dt = 2024-09-02 (the date yielding the lowest uplift) was specifically chosen to provide a conservative, worst-case assessment when comparing the performance differences across these different horizons.

A longer simulation horizon (e.g., 12 weeks) allows the optimizer to internalize the full lifecycle of demand, returns, and replenishment cycles. This enhances strategic planning and mitigates long-term overstock and fulfillment costs by capturing delayed effects such as product returns and season-end demand shifts. Conversely, shorter horizons (e.g., 6 weeks) prioritize agility and responsiveness, focusing on immediate demand signals but limiting the model’s capacity to anticipate downstream inventory imbalances.

The exponential decay weighting amplifies this dynamic by assigning greater importance to early-period costs (e.g., holding or lost sales), effectively discounting the influence of later weeks. As a result, longer horizons appear more cost-efficient, since late-period fluctuations contribute less to the objective function, while shorter horizons emphasize service reliability at the expense of long-term cost minimization.

Overall, this interaction illustrates a classical trade-off in inventory optimization: shorter horizons enhance responsiveness and service performance, whereas longer horizons promote profitability and cost stability through lifecycle-aware planning.

| Simulation Horizon | GMV Uplift wrt Human Baseline | Uplift of GMV after fulfilment costs wrt Human Baseline | % Merchants with  Positive GMV Uplifts | Weekly availability rate of the model | Demand fill rate of the model | Weekly Availability rate uplift wrt Human | Demand  fill rate uplift  wrt Human |
| --- | --- | --- | --- | --- | --- | --- | --- |
|  |  |  |  |  |  |  |  |
| **12 weeks**  **(ours)** | 14.11%  [8.48%, 23.47%] | 13.23%  [7.24%, 23.32%] | 73.61%  [69.86%, 77.05%] | 86.08  [85.23, 86.86] | 90.91  [90.44, 91.41] | +32.44% [+29.05%, +35.86%]  +21.08pp [+19.37, +22.74] | +24.20% [+21.69%, +26.54%]  +17.71pp [+16.38, +19.13] |
| **8 weeks** | 11.85%  [7.49%, 18.53%] | 11.85%  [7.57%, 18.36%] | 65.58% [61.39%, 69.53%] | 84.46  [83.41, 85.52] | 90.30  [89.62, 90.96] | +26.36% [+23.28%, +29.72%]  +17.62pp [+15.99, +19.29] | +19.62% [+17.62%, +21.74%]  +14.81pp [+13.48, +16.12] |
| **6 weeks** | 6.68%  [2.85%, 12.91%] | 6.73%  [2.38%, 13.00%] | 63.53%  [59.27%, 67.59%] | 79.57  [78.26, 80.86] | 86.77  [85.94, 87.64] | +18.88% [+16.05%, +21.95%]  +12.64pp [+11.06, +14.29] | +14.79% [+12.79%, +16.84%]  +11.18pp [+9.77, +12.46] |

Table 8. Simulation-Horizon Sensitivity (6, 8, and 12 weeks). Effect of optimization horizon length on financial and operational outcomes of the Replenishment Engine. All results are reported for exec_dt = 2024-09-02, the least-uplift date within the October 2023 – September 2024 backtest, to highlight robustness under conservative conditions. Metrics include GMV uplift, GMV after fulfillment costs uplift, proportion of merchants with positive GMV uplifts, weekly availability, demand fill rate, and corresponding uplifts versus human benchmarks. Bracketed values represent 95 % confidence intervals.

**Cost Percentile Sensitivity**

| Cost Percentile | GMV Uplift wrt Human Baseline | Uplift of GMV after fulfilment costs wrt Human Baseline | % Merchants with  Positive GMV Uplifts | Weekly availability rate of the model | Demand fill rate of the model | Weekly Availability rate uplift wrt Human | Demand  fill rate uplift  wrt Human |
| --- | --- | --- | --- | --- | --- | --- | --- |
|  |  |  |  |  |  |  |  |
| **75th percentile**  **(ours)** | 14.11%  [8.48%, 23.47%] | 13.23%  [7.24%, 23.32%] | 73.61%  [69.86%, 77.05%] | 86.08  [85.23, 86.86] | 90.91  [90.44, 91.41] | +32.44% [+29.05%, +35.86%]  +21.08pp [+19.37, +22.74] | +24.20% [+21.69%, +26.54%]  +17.71pp [+16.38, +19.13] |
| **50th percentile** | 11.74%  [8.21%, 17.21%] | 12.54% [8.31%, 18.27%] | 61.79%  [57.69%, 65.72%] | 79.86  [78.68, 81.01] | 86.28 [85.50, 87.13] | +18.80% [+16.16%, +21.80%]  +12.64pp [+11.07, +14.33] | +15.24% [+13.23%, +17.28%]  +11.41pp [+10.13, +12.78] |
| **90th percentile** | 16.06%  [11.91%, 22.19%] | 16.98% [12.09%, 23.14%] | 66.13%  [62.13%, 69.92%] | 82.78  [81.71, 83.81] | 88.39  [87.63, 89.11] | +22.69% [+19.98%, +25.71%]  +15.31pp [+13.79, +16.87] | +17.82% [+15.78%, +20.01%]  +13.37pp [+12.12, +14.62] |

Table 9: Cost Percentile Sensitivity. Effect of optimizing different cost percentiles (risk tolerance) on financial and operational outcomes of the Replenishment Engine. All results are for exec_dt = 2024-09-02.

Table 9 examines the sensitivity of financial and operational outcomes to the cost-percentile objective, which governs the model’s effective risk tolerance. Moving from the 50th to the 90th percentile corresponds to a transition from risk-neutral to risk-averse optimization behavior. As the percentile increases, both GMV uplift and service levels (availability and fill rate) generally improve, indicating that greater risk aversion enhances operational stability. However, the operational uplifts for availability (+21.08pp) and fill rate (+17.71pp), as well as the proportion of merchants with positive GMV uplifts (73.61%), peak at the 75th percentile. This suggests that performance gains in service levels plateau beyond the 75th percentile, indicating diminishing returns from excessive conservatism.

Our choice of the 75th percentile for cost optimization is grounded in four complementary rationales from the operations research and supply chain management literature. First, following Rockafellar, R. T., & Uryasev, S. (2000), optimizing higher percentiles provides a computationally tractable approximation to Conditional Value-at-Risk (CVaR), which is particularly suitable for inventory systems where tail risks have asymmetric business impacts—the consequences of stockouts typically far exceed those of moderate overstocks. Second, consistent with Silver, E. A. (2016), industry-standard inventory systems typically target service levels between 75–95%, making a 75th-percentile cost optimization naturally aligned with real-world B2B service expectations. Third, Schweitzer, M. E. (2000) provide behavioral evidence that supply chain decision-makers act as if optimizing approximately the 70–80th percentile of outcome distributions, reflecting realistic managerial risk preferences rather than purely expected-value logic. Finally, Bertsimas, D. (2006) show that percentiles in the 75–85% range offer the optimal balance between risk protection and numerical stability in finite-sample stochastic programs, whereas higher percentiles may lead to unstable or overly conservative solutions within our 500-simulation Monte Carlo framework.

Taken together, these theoretical and empirical insights justify the 75th-percentile objective as a theoretically sound, behaviorally consistent, and computationally stable risk threshold. It achieves the most favorable trade-off between profitability, service-level reliability, and robustness, ensuring consistent decision quality under uncertainty in e-commerce replenishment systems.

# References

1. **Bertsimas, D. (2006)**. *Robust optimization*.
2. **Rockafellar, R. T., & Uryasev, S. (2000)**. Optimization of Conditional Value-at-Risk. *Journal of Risk, 2*(3), 21–41.
3. **Schweitzer, M. E. (2000)**. *Judgment and decision making in organizations*.
4. **Silver, E. A. (2016)**. *Inventory management and production planning and scheduling*.
